# lnc-REG3G-3-1/miR-215-3p Promotes Brain Metastasis of Lung Adenocarcinoma by Regulating Leptin and SLC2A5

**DOI:** 10.3389/fonc.2020.01344

**Published:** 2020-08-12

**Authors:** Chunyang Jiang, Hui Zhao, Bingjun Yang, Zengfeng Sun, Xin Li, Xiaoli Hu

**Affiliations:** ^1^Department of Thoracic Surgery, Tianjin Union Medical Center, Nankai University, Tianjin, China; ^2^Key Laboratory of Cancer Prevention and Therapy, Department of Neurosurgery and Neurooncology, National Clinical Research Center for Cancer, Tianjin Medical University Cancer Institute and Hospital, Tianjin, China; ^3^Department of Thoracic Surgery, Tianjin Chest Hospital, Tianjin, China; ^4^Department of Respiratory, The Second People's Hospital of Linhai City, Taizhou, China

**Keywords:** lncRNA, microRNA, lung adenocarcinoma, brain, metastases

## Abstract

This study aims to explore the role and mechanism of specific lncRNA in brain metastasis (BM) from lung adenocarcinoma (LADC), providing an effective biomarker for early diagnosis and targeted therapy of BM from LADC. Based on the gene expression profiles of lncRNA and mRNA in LADC and BM tissues detected by Gene Chip, lnc-REG3G-3-1 was selected, and the related genes, including *miR-215-3p, leptin*, and *SLC2A5*, were identified by data analysis. Human LADC cell lines A549 and H1299 were cultured. Dual-luciferase and endogenous validation experiments were used to confirm the regulation between these genes. Real-time quantitative reverse transcription–polymerase chain reaction and Western blotting were used to detect gene expression. The tumor metastasis-related gene function of lnc-REG3G-3-1 and miR-215-3p in H1299 cells was verified by Transwell invasion, migration assays, and scratch testing. Nude mice xenograft tumors constructed with decreased lnc-REG3G-3-1 confirmed the influences on gene expression *in vivo*. lnc-REG3G-3-1 was highly expressed in BM tissues that originated from LADC compared with that in primary cancer tissues. lnc-REG3G-3-1 reduced miR-215-3p expression, thereby regulating the target genes *leptin* and *SLC2A5* and the signaling pathways, taking part in the lnc-REG3G-3-1/miR-215-3p axis in the process of BM from LADC. lnc-REG3G-3-1, leptin, and SLC2A5 through regulating signaling pathways may be jointly involved in the regulation of the biological process of BM in patients with LADC.

## Introduction

The morbidity and mortality of lung cancer are highest among malignant tumors in some large cities of industrialized countries (1, 2). Non-small cell lung cancer (NSCLC) accounts for about 85% of lung cancer (3), and adenocarcinoma is the most common histological type, accounting for about 35% of lung cancer cases, which is more prone to blood and lymphatic metastasis (4). Metastasis is a malignant sign and characteristic of lung cancer and also a major cause of treatment failure and patient death (5). The early diagnosis of lung cancer metastasis is very important for improving the treatment level and prognosis of patients.

Although current studies have found that multiple mechanisms are involved in the metastasis process of lung cancer (6–9), the molecular mechanism of lung cancer metastasis is still not completely clear. Therefore, elucidating the molecular mechanism of lung cancer metastasis can better understand the complex biological behavior of lung cancer and search for molecular markers or targets that are sensitive to the early diagnosis and treatment of lung cancer metastasis to provide a scientific basis for preventing or delaying the occurrence and development of metastasis and effectively improving the prognosis of patients.

The high incidence of brain metastases (BMs) in lung adenocarcinoma (LADC) often lacks effective treatment. It is an important cause of treatment failure and patient death (10). Patients with BMs experience poor prognosis and significant morbidity, often with a median survival time of 3–6 months (11). The process of BMs from LADC in specific mechanism studies is not enough. The lack of an effective early diagnosis method of BMs from LADC results in some patients already with distant metastasis when seeing a doctor or in the process of treatment delays the optimal timing of treatment. This would seriously influence the quality of life and long-term survival of patients with LADC.

Long non-coding RNA (lncRNA) accounts for 80% of the total amount of all non-coding RNA (ncRNA), which can regulate gene expression on multiple levels, participating in almost all physiological and pathological processes of organisms (12). The abnormal expression of lncRNA in tissues is directly related to the process of carcinogenesis. LncRNA is abnormally expressed in a variety of human tumors and shows the specificity of tumor tissue, which can regulate the biological function of specific tumor cells and is considered as a new specific tumor marker (13). More and more recent research results indicate that lncRNAs are becoming the main regulatory factors of tumor cell phenotype, involving oncogenes, and tumor suppressor genes (14). Some lncRNAs are closely related to the invasion and metastasis of malignant tumors (15). It is believed that, with the deepening of studies on the biological function of lncRNA, more lncRNAs related to tumor metastasis will be found, which will surely provide new clues for the diagnosis and treatment of tumors.

This study aims to find the specific lncRNA molecule relating BMs from LADC *in situ* tumor to BM tumor tissue using gene chip technology and, combined with the biological information database, through the competitive endogenous RNA (ceRNA) mechanism for microRNA (miRNA) related to specific lncRNA to verify the possible target genes and, therefore, to explore the role and mechanism of specific lncRNA in BMs from LADC. These findings provide a scientific basis for the early diagnosis and targeted therapy of BMs from LADC for application in further studies.

## Materials and Methods

### Main Reagents

Human LADC cell lines A549 and H1299 and human lung normal lung epithelial cell line BEAS-2B were obtained from the Shanghai Cell Bank of the Chinese Academy of Sciences (Shanghai, China). Dulbecco's modified Eagle's medium (DMEM), Opti-MEM, and fetal bovine serum (FBS) were obtained from Gibco (USA). The RNAprep Pure Cell/Bacteria Kit was purchased from Tiangen Biotech (Beijing, China). Trizol reagent was purchased from Invitrogen (USA). The Revert Aid First Strand cDNA Synthesis Kit was purchased from Fermentas (Lithuania). SYBR Prime Script RT-PCR Kit and Thermal Cycler Dice™ Real Time System III were purchased from TaKaRa (Japan). Lipofectamine 2,000 was purchased from Invitrogen. The B-type plasmid small quantity rapid extraction kit was purchased from BioDev-Tech Scientific & Technical Co., Ltd. (Beijing, China). Restriction endonuclease and Trans2K Plus II DNA Marker were purchased from Thermo, Inc. (USA). The dual-luciferase reporter gene assay kit was purchased from the Beyotime Institute of Biotechnology (Haimen, China). The 3-(4,5-dimethylthiazol-2-yl)-2,5-diphenyltetrazolium bromide (MTT) assay kit was purchased from Ameresco (Cincinnati, OH, USA). Matrigel and Transwell chambers were purchased from Becton-Dickinson Biosciences (San Jose, CA, USA).

miRNA mimics were obtained from GenePharma, Inc. (Shanghai, China). pmirGLO plasmid was constructed and obtained from Tianjin Saierbio Technology, Inc. (China). Antibodies specific to leptin, SLC2A5, JAK2, Stat3, vascular endothelial growth factor (VEGF), FLAD1, STYXL1, and glyceraldehyde 3-phosphate dehydrogenase (GAPDH) were purchased from Santa Cruz Biotechnology (Santa Cruz, CA, USA). Phospho-JAK2 (p-JAK2) and phospho-Stat3 (p-Stat3) antibodies were purchased from Cell Signaling Technology, Inc. (USA). Goat anti-rabbit IgG coupled to horseradish peroxidase (HRP) was purchased from Pierce (Rockford, IL, USA). All other reagents used were analytical-grade laboratory chemicals from standard commercial suppliers.

### Patients and Samples

Five paired cancer and adjacent normal tissues from five patients with lung cancer were randomly collected. All patients had surgically proven primary LADC and received pulmonary lobectomy at the Tianjin Union Medical Center (TUMC) between October 2016 and January 2017. Three paired cancer and adjacent normal tissues from three patients with BMs were randomly collected. All three patients had surgically proven BMs from LADC and received BM tumor resection at the Tianjin Medical University Cancer Institute and Hospital between October 2017 and March 2018. All samples were obtained with informed consent and underwent examination for the lncRNA expression profile of the tissues by gene chip detection. The details of the clinical and pathological information of these patients are shown in Tables 1, 2. The study was approved by the Medical Ethics Committee of the TUMC.

**Table 1:** Clinical characteristics of patients with lung adenocarcinoma (*n* = 5).

**Patients**	
**Clinical factors**	**Data**
Age (years)	65.4 (mean)
Gender (Male/Female)	2/3
Smoking (Y/N)	2/3
Single or multiple	S
Tumor diameter (cm)	3.42 (mean)
Histology	LAD

**Table 2:** Clinical characteristics of patients with lung adenocarcinoma and brain metastases of lung adenocarcinoma (*n* = 3).

**Clinical factors**	**Patients**		
	**No 1**	**No 2**	**No 3**
Age (years)	61	52	58
Gender	F	F	M
Smoking	N	N	N
Tumor site in brain	Cerebellum	Occipital lobe/parietal lobe	Occipital lobe
Single or multiple	Multiple	Two sites	Single
Tumor volume (cm)	4.0 × 3.0 cm	4.0 × 3.2 cm/5.2 × 3.4 cm	5.0 × 4.0 cm
Histology	Adenocarcinoma	Adenocarcinoma	Adenocarcinoma

### Tissue Sample Gene Chip Detection

CapitalBio Technology lncRNA + mRNA Human Gene Expression Microarray version 4.0 (CapitalBio Corp., Beijing, China) was used to detect lncRNA and mRNA expression between LADC and paracancerous tissues. The lncRNA and mRNA expression of BMs and paracancerous tissue samples of LADC were also detected using this. The test method number was AG-GE-WL10-01-2010, and the data analysis method number was AG-GE-DL00-01-2010.

### RNA Extraction, Amplification, Labeling, and Array Hybridization

Total RNA was first extracted from the specimens using Trizol reagent and purified with mirVana miRNA Isolation Kit (Ambion, Austin, TX, USA) according to the manufacturer's protocol. cDNA labeled with a fluorescent dye (Cy3-dCTP) was produced by the Eberwine's linear RNA amplification method and subsequent enzymatic reaction. The procedure was referenced as described previously (16), and the procedure was improved using CapitalBio cRNA Amplification and Labeling Kit for producing higher yields of labeled cDNA. The cRNA amplification and labeling procedure was depicted as described previously (17).

### Microarray Imaging and Data Analysis

The lncRNA + mRNA array data were analyzed for data summarization, normalization, and quality control using GeneSpring software version 13.0 (Agilent). Threshold values of ≥2- and ≤ 2-fold change and a corrected *P* = 0.05 were used to select differentially expressed genes. Data were log_2_ transformed using the Adjust Data function of CLUSTER 3.0 software and further analyzed by hierarchical clustering with average linkage.

### Cell Culture

Human LADC cell lines A549 and H1299 were selected and cultured in DMEM containing L-glutamine (2 mM), 10% FBS, and 1% penicillin and streptomycin (100 units/mL and 100 μg/mL, respectively), in an incubator at 37°C with 95% humidified air and 5% CO_2_. The human lung normal lung epithelial cell line BEAS-2B was selected as control and cultured using the same methods.

### Real-Time Quantitative Reverse Transcription–Polymerase Chain Reaction (RT-PCR)

After cell collection, total cellular RNA was isolated. A total of 2.0 μg RNA was reverse transcribed to cDNA using Revert Aid First Strand cDNA Synthesis Kit. All primers were designed and synthesized by the General Biosystems, Inc. (Anhui, China). For primer sequences for miRNAs, lnc-REG3G-3-1, leptin, SLC2A5, and β-actin, see Supplementary Table 1; for primer sequences for lnc-REG3G-3-1-3′UTR-wt and lnc-REG3G-3-1-3′UTR-mut, see Supplementary Table 2; for primer sequences for pshR-lnc-REG3G-3-1, see Supplementary Table 3; and for primer sequences for Stat3, mammalian target of rapamycin (mTOR), JAK2, STYXL1, VEGF, AKT, Notch-1, phosphatidylinositol 3-kinase (PI3K), CUL7, FLAD1, and SOX4, see Supplementary Table 4. Real-time RT-PCR was used with the SYBR Premix Ex Taq kit and detected with Thermal Cycler Dice™ Real-Time System III. The housekeeping gene was validated by agarose gel electrophoresis and β-actin was selected and used as an internal control in parallel for each trial. The fluorescence threshold value was calculated using SDS 2.2.1 system software, and the ^2−ΔΔ^Ct method was applied for quantitative calculation. All experiments were performed three times independently, and the average was used for comparison.

### RNA Fluorescence *in situ* Hybridization (FISH)

H1299 cells (1 × 10^5^) were inoculated in 24-well cell culture plates (with a glass slice at the bottom) and placed in an incubator at 37°C, 5% CO_2_ for 24 h. The glass slices with cell growth on the surface were taken out and fixed with 4% paraformaldehyde at room temperature for 30 min. The fixed liquid was removed and washed with phosphate-buffered saline (PBS) twice. Slices were digested by a dropwise addition of 3% pepsin solution (1 mL) and blended for 2 min at 37°C. Cells were washed, and prehybridization solution (20 μL) was added and incubated. After hybridization, SSC buffer was used to wash the cells. Cells were dehydrated and dried in air. Each well was added with 300 μL 4′,6-diamidino-2-phenylindole (1 μg/mL) for 5 min. The liquid was removed and washed with precooled PBS. Cells on the glass slices were observed and photographed under a fluorescence microscope.

### Target Gene Verified by Dual-Luciferase Method

After H1299 cells were transfected, dual-luciferase reporter gene assay was performed according to the kit. The report gene cell lysis fluid was used as a blank control. After completing the determination of firefly luciferase, 100 μL *Renilla* luciferase assay solution was added and homogenized, and RLU was determined by GloMax 96 microporous plate luminescence detector. The internal reference was *Renilla* luciferase. The RLU value determined by firefly luciferase was divided by the RLU value determined by *Renilla* luciferase. The degree of gene activation was compared between different samples according to the ratio obtained.

### Cell Transfection

The H1299 cell suspension was drained, and 1 × 10^5^ cells were inoculated in a six-well cell culture plate in an incubator at 37°C, 5% CO_2_ for 24 h. Cell transfection solutions A and B were prepared in a sterilized 1.5 mL centrifuge tube. Liquid A was obtained by diluting 6 μL transfection reagent with serum-free DMEM to a final volume of 50 μL. Liquid B was obtained by diluting 3 μL Lipofectamine 2,000 reagent with serum-free DMEM to a final volume of 50 μL. Solutions A and B were gently mixed and placed at room temperature for 5 min. The mixing transfection solution and Opti-MEM were added to each well of the cell culture plate, and the transfected cells were cultured in an incubator at 37°C, 5% CO_2_.

### Western Blotting and MTT Assay

Western blotting and MTT assay were performed as described in our previous studies (18, 19).

### Transwell Invasion and Migration Experiments

Each Transwell chamber was coated with 10 μL fibronectin (0.5 mg/mL) and dried to solidify fibronectin at the bottom of the membrane. Dissolved matrix glue was added at 50 μL/well. At the same time, transfected H1299 cells were digested and counted. Then, 10^5^ cells were placed into a 1.5 mL EP tube and centrifuged at 2,000 rpm for 5 min, the supernatant was removed, and the suspended cells were added into the Transwell chamber with 200 μL serum-free DMEM. DMEM containing 20% serum (Gibco) was added to the lower chamber. Cells were incubated at 37°C for 24 h. The Transwell chamber was removed, and the cells were gently washed with PBS. The mixed solution was prepared with methanol/glacial acetic acid (3:1), and the cells on the opposite side of the Transwell chamber were fixed for 30 min and then added into crystal violet dye solution, stained for 15 min, and cleaned. The membrane was fixed on the glass slide for observation.

### Scratch Test

The ability of cell migration was measured by the scratch test. The transfected H1299 cells were cultured in a 24-well plate until the density was close to the fusion state. The tip of the add sampler was used against the ruler as perpendicular as possible to the horizontal line behind the plates to perform the scratch test. The cells were washed with PBS three times to remove the subtracted cells, and serum-free medium was added. The cells were added to the culture plate in an incubator at 37°C, 5% CO_2_. Samples were taken at 0, 24, and 48 h, and photos were taken under the microscope.

### Xenograft Tumors in Nude Mice

Female BALB/c nude mice (8 weeks old) were purchased from the Experimental Animal Center of the Chinese Academy of Sciences. The experimental protocols were approved by the ethics committee of TUMC. The animals were divided into three groups: blank H1299 control group, pSilencer-NC group, and pshR-REG3G-3-1 group, with six nude mice in each group. The screened stable H1299-pSilencer-NC and H1299-pshR-REG3G-3-1 cells were amplified and cultured, respectively. Then, a 1-mL syringe was used to absorb the cell suspension by emptying bubbles and injected subcutaneously into the right back of nude mice. The control group was injected with H1299 cells. The transfection control group was injected with tumor cells H1299-pSilencer-NC. The transfection group was injected with tumor cells H1299-pshR-REG3G-3-1. The weight of nude mice was measured before inoculation, and the tumor could be felt under the skin (~12 days). The body weight of each animal was weighed every 5 days. Tumor length and short diameters were measured every 3 days to calculate the tumor volume. The average tumor volume of each group was used to plot the growth curve of the transplanted tumors. After 22 days, nude mice were euthanized with ether anesthesia and photographed. The tumor was completely removed, weighed, and photographed (with scale). The tumor samples were divided into two parts: one was fresh tumor tissue stored at −80°C for the detection of gene and protein expression, and the other was placed in neutral formalin for fixation for immunohistochemical examination as described previously (20).

### Statistical Analysis

All data were analyzed using two-way univariate analysis of variance followed by Tukey's (equal variances assumed or homogeneity of variance after variable transformation) or Dunnett's T3 (equal variances not assumed after variable transformation justification) *post-hoc* test between groups using the Statistical Package for Social Sciences software version 20.0 (SPSS, Chicago, IL, USA). Results were taken as mean ± SD. All tests were two sided. *P* < 0.05 was considered statistically significant.

## Results

### Differential Expression of lncRNAs and mRNAs in Cancer Tissues

By detecting the gene expression profiles of lncRNA and mRNA in cancer tissues of five cases of LADC and the paired paracancerous tissue samples, it was found that there were a large number of differentially expressed lncRNAs and mRNAs in LADC tissues and paracancerous tissues. The expression differences of lncRNA and mRNA are listed in Supplementary Table 5 [total 20,179, shown are FC (abs) more than 2.0 times] for LADC lncRNA and Supplementary Table 6 [total 25,154, shown are FC (abs) more than 2.0 times] for LADC mRNA. The detection results of the expression profiles of gene groups in heat maps are shown in Supplementary Figure 1A.

By detecting the gene expression profiles of lncRNA and mRNA in three cases of BMs from LADC and matched paracancerous tissues, it was found that there were significant differences in the expression of lncRNAs and mRNAs between BM tumors of LADC and paracancerous tissues. The differences between lncRNA and mRNA expression are listed in Supplementary Table 7 for lncRNA and Supplementary Table 8 for mRNA. The genetic heat maps of the test results are shown in Supplementary Figure 1B. All these data are provided by CapitalBio for detection and analysis.

### Combined Enrichment Gene Microarray in Tissues of LADC and BM Tumors From LADC

In order to find whether there exist key genes related with LADC and BMs, the combined analysis of the lncRNA expression difference between BMs and LADC tissues (T vs. C) of these patients were performed, and the results are listed in Supplementary Table 9 [total 26,642, shown are FC (abs) more than 2.0 times]. According to the relationship between lncRNAs and signaling pathway genes, the enrichment analysis results of the signal transduction pathway involving the first 10 lncRNAs in *P*-value sequencing showed that the lncRNAs related to NSCLC presented significant enrichment (Supplementary Figure 2). The significant differences in lncRNA expression (top 8) between LADC and BMs from LADC are listed in Supplementary Table 10. Two lncRNAs were significantly upregulated, including ENST00000439259.1 (lnc-REG3G-3-1), and six lncRNAs were significantly downregulated. For the two upregulated lncRNAs, the shorter lnc-REG3G-3-1 was first selected to performed confirmatory study. The RNA-protein interaction prediction (RPISeq) database was used to analyze and predict the interaction between lnc-REG3G-3-1 and target genes. The results showed that the RF classifier and SVM classifier values of lnc-REG3G-3-1 and leptin and SLC2A5 were <0.5 (0.83, 0.89; 0.75, 0.81, respectively), indicating a strong interaction relationship. Standalone BLAT v.35 was used to predict lnc-REG3G-3-1 and target mRNAs, and the results showed that lnc-REG3G-3-1 could regulate the expression of target genes leptin and SLC2A5 through ceRNA regulation. Lnc-REG3G-3-1 reduced the expression level of a specific miRNA in miR-215-3p, miR-4505, and miR-5787 through the adsorption by ceRNA mechanism, thus regulating the expression of target genes leptin and SLC2A5.

### Effect of lnc-REG3G-3-1 and miR-215-3p on Cellular Functions

**Figure 1:**
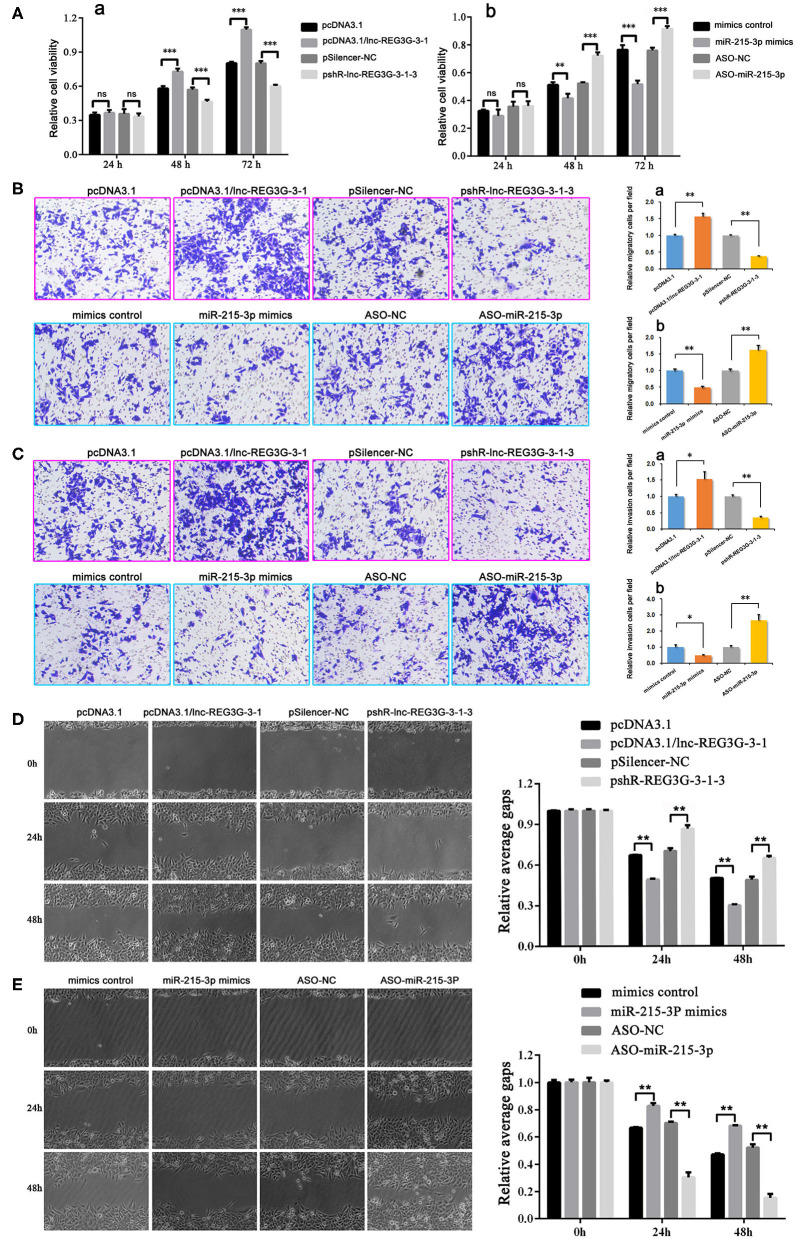
Cellular functions of lnc-REG3G-3-1 and miR-215-3p in H1299 cells. After transfection with plasmids, the expression of lnc-REG3G-3-1 and miR-215-3p were regulated. After cells were cultured for 24, 48, and 72 h, MTT assay was used to detect H1299 cell viability **(A**,a,b**)**. After transfection with plasmids to regulate the expression of lnc-REG3G-3-1 and miR-215-3p for 24 h, Transwell invasion and Transwell migration assays were used to detect the differences of migration and invasion abilities of H1299 cells **(B,C)**. The results of cell migration detected by scratch testing were observed under a light microscope, and the results show that lnc-REG3G-3-1 and miR-215-3p influence cell migration abilities **(D,E)**. Bar graphs represent the ratios plotted as mean ± SD of three separate experiments. Compared with the control group, “ns” means no statistical difference between means, *P < 0.05; **P < 0.01; ***P < 0.001.

The MTT assay was used to detect the viability of H1299 cells. The results showed that, at 48 and 72 h, overexpressed lnc-REG3G-3-1 can significantly increase H1299 cell viability compared with the control group. After the knockdown of lnc-REG3G-3-1, the cell activity was significantly lower than that of the control group (Figure 1A,a). When miR-215-3p was overexpressed and cultured for 48 and 72 h, cell viability was significantly decreased compared with that of the control group, whereas with the knockdown of miR-215-3p and culture for 48 and 72 h, cell viability was significantly increased compared with the control group (Figure 1A,b).

The transwell invasion and migration assays combined with the scratch test were employed to detect the changes in the ability of cells to invade and migrate induced by regulation of lnc-REG3G-3-1 or miR-215-3p expression. The results showed that, compared with the control group, the overexpression of lnc-REG3G-3-1 can significantly enhance the migration and invasion abilities of cells, whereas the knockdown of lnc-REG3G-3-1 can significantly reduce the migration and invasion abilities of cells (Figures 1B–D). In contrast, the overexpression of miR-215-3p significantly reduced the abilities of cell migration and invasion compared with the control group. However, the knockdown of miR-215-3p significantly increased the migration and invasion abilities of cells compared with the control group (Figures 1B,C,E).

### Expression Differences of lnc-REG3G-3-1, Leptin, and SLC2A5 Genes and miRNAs

**Figure 2:**
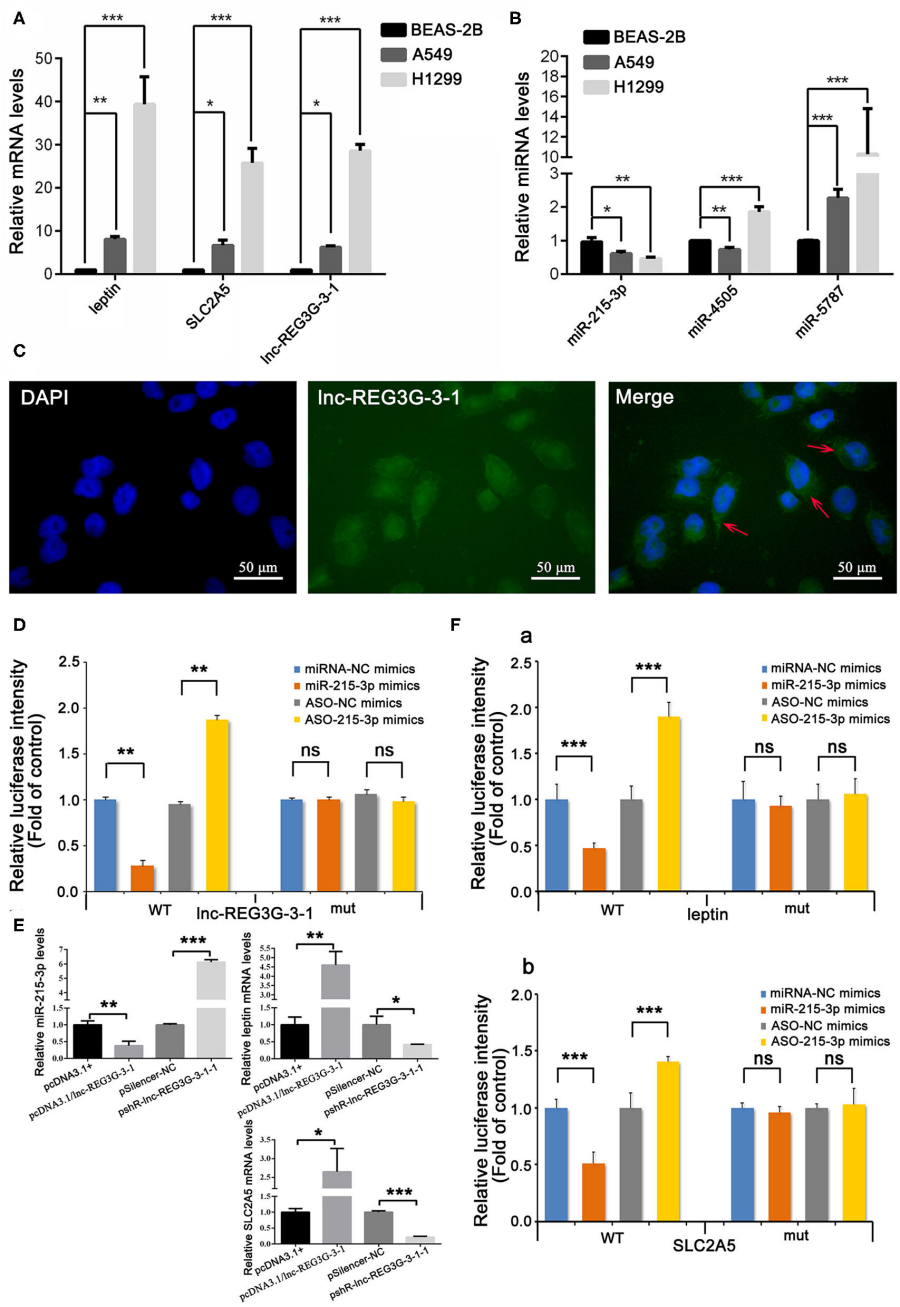
Differential expression of lnc-REG3G-3-1, miR-215-3p, leptin, and SLC2A5 and miRNAs in lung cancer cell lines and cellular localization of lnc-REG3G-3-1. Real-time RT-PCR was employed to confirm the expression differences of the gene levels of lnc-REG3G-3-1, leptin, and SLC2A5, and three miRNAs, including miR-215-3p, miR-5787, and miR-4505 in A549 and H1299 cells and the BEAS-2B cell line as control. The results are shown in **(A,B)**. RNA FISH assay was used to detect the expression and localization results of lnc-REG3G-3-1 in H1299 cells **(C)**. The abundant expression of lnc-REG3G-3-1 in the cytoplasm is pointed out by red arrows in the merged image. Dual-luciferase reporter assay was used to verify whether lnc-REG3G-3-1 has a specific adsorption effect on miR-215-3p in H1299 cells **(D)**. The effects of the overexpression and knockdown of lnc-REG3G-3-1 on the expression levels of miR-215-3p, leptin, and SLC2A5 in H1299 cells were detected by real-time RT-PCR **(E)**. Dual-luciferase reporter assay was used to verify whether miR-215-3p has a targeting effect on leptin and SLC2A5 in H1299 cells. The results showed that miR-215-3p has a targeting effect on leptin **(F**,a**)** and also with the same function on the SLC2A5 gene **(F**,b**)**. Bar graphs represent the ratios plotted as mean ± SD of three separate experiments. Compared with the control group, *P < 0.05; **P < 0.01; ***P < 0.001.

Based on these bioinformatics results, real-time RT-PCR was used to detect the expression differences of lnc-REG3G-3-1, leptin, and SLC2A5 genes and the three miRNAs in A549 and H1299 cells and human normal lung epithelial cell line BEAS-2B as control. The results show that the expression of lnc-REG3G-3-1, leptin, and SLC2A5 in A549 and H1299 cells was significantly increased compared with that in control cells, and the expression in H1299 cells was significantly increased compared with that in A549 cells (Figure 2A). The expression of miR-215-3p was significantly decreased in both A549 and H1299 cells, and the expression of miR-5787 was significantly increased. miR-4505 was decreased in A549 cells and increased in H1299 cells (Figure 2B).

### Expression and Localization of lnc-REG3G-3-1 in H1299 Cells

The subcellular localization of lncRNA is closely related to its mechanism of action. The RNA FISH assay is an important method for lncRNA subcellular localization, which was employed in the present study to detect the expression and localization of lnc-REG3G-3-1 in H1299 cells. H1299 cells were transfected with the FITC probe, which was constructed and labeled by lnc-REG3G-3-1 FISH to observe the relative localization and expression of lnc-REG3G-3-1 in H1299 cells. The results showed that lnc-REG3G-3-1 had abundant expression in the cytoplasm (Figure 2C).

### Confirmation of miR-215-3p as a Specific Adsorption Target Gene of lnc-REG3G-3-1

Luciferase reporter gene assays using luciferin as the substrate to detect the activity of firefly luciferase are widely used for miRNA target gene validation. In the present study, dual-luciferase reporter assay was used to verify whether lnc-REG3G-3-1 has a specific adsorption effect on miR-215-3p in H1299 cells. The results show that luciferase activity decreased after the overexpression of miR-215-3p and increased after the knockdown of miR-215-3p. After mutation of lnc-REG3G-3-1, miR-215-3p did not affect luciferase activity. The results confirm that lnc-REG3G-3-1 had a specific adsorption effect on miR-215-3p (Figure 2D).

### Endogenous Verification That lnc-REG3G-3-1 Affected the Expression of miR-215-3p, Leptin, and SLC2A5

By transfecting the recombinant plasmid pcDNA3.1/lnc-REG3G-3-1 and pshR-lnc-REG3G-3-1, the effects of the overexpression and knockdown of lnc-REG3G-3-1 on the expression levels of miR-215-3p, leptin, and SLC2A5 in H1299 cells were detected by real-time RT-PCR. The results show that the overexpression of lnc-REG3G-3-1 inhibited the expression of miR-215-3p, and the knockdown promoted its expression, further verifying the targeted adsorption effect of lnc-REG3G-3-1 on miR-215-3p. The overexpression of lnc-REG3G-3-1 promoted the expression of leptin and inhibited the expression of leptin after knockdown. The overexpression of lnc-REG3G-3-1 promoted the expression of SLC2A5 and inhibited its expression after knockdown (see Figure 2E).

### Verification of the Targeting Effects of miR-215-3p on Leptin and SLC2A5

The dual-luciferase reporter assay results show that, when miR-215-3p was overexpressed, luciferase activity decreased, whereas the knockdown of miR-215-3p increased luciferase activity. After leptin-3′UTR mutation, the changes in miR-215-3p did not affect the activity of luciferase. The results show that miR-215-3p has a targeting effect on leptin (Figure 2F,a). After SLC2A5-3′UTR mutation, the changes in miR-215-3p did not affect the activity of luciferase. The results show that miR-215-3p has a targeting effect on SLC2A5 (Figure 2F,b).

### Effect of miR-215-3p on mRNA Expression of Genes Related to Leptin and SLC2A5 Signaling Pathway

**Figure 3:**
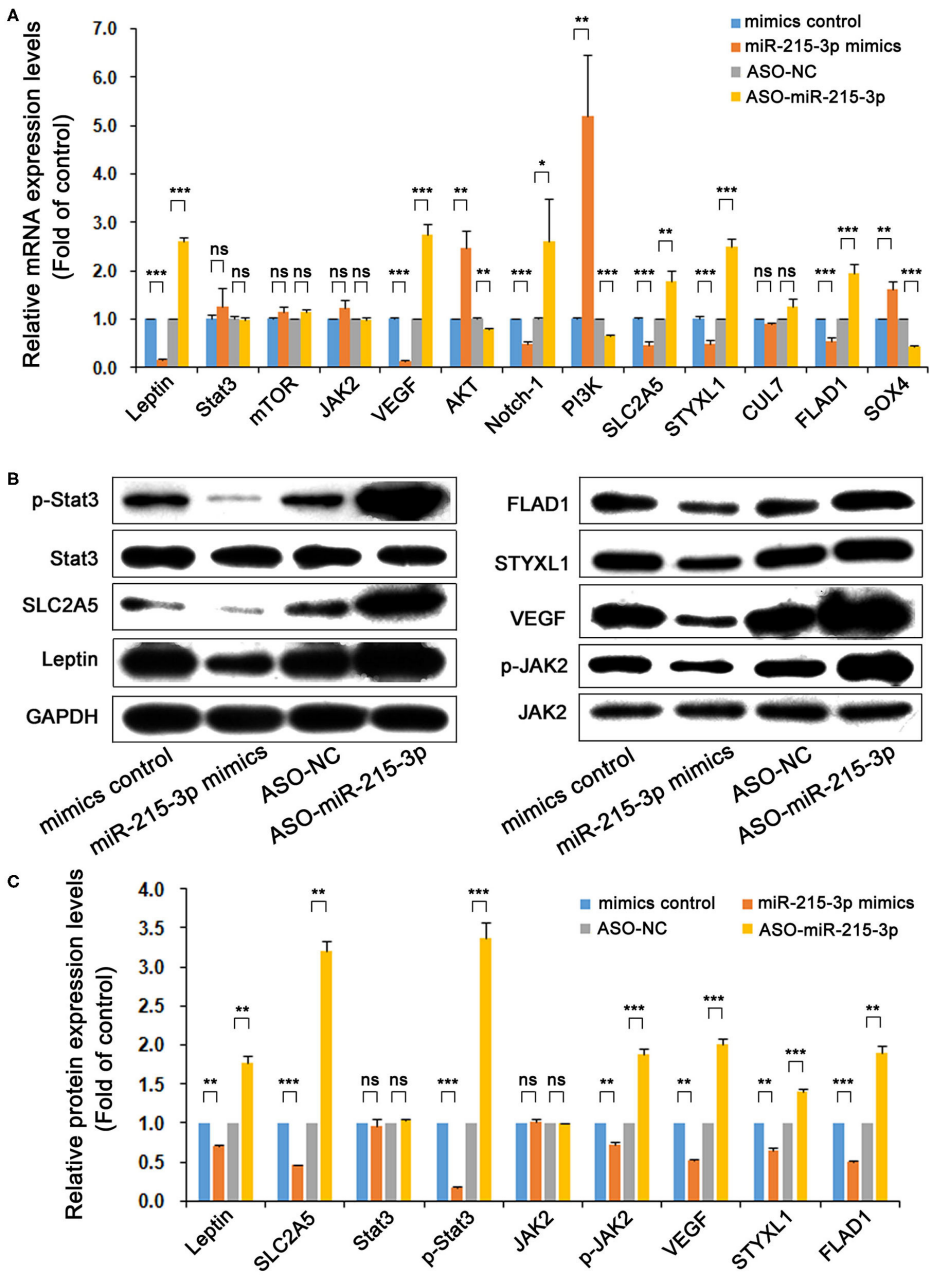
miR-215-3p effect on mRNA and protein expression of genes related to leptin and SLC2A5 signaling pathway. After the overexpression or knockdown of miR-215-3p by transfected plasmids, the mRNA expression levels of leptin, SLC2A5, Stat3, mTOR, JAK2, VEGF, AKT, Notch-1, PI3K, STYXL1, CUL7, FLAD1, and SOX4 were detected by real-time RT-PCR **(A)**. Upregulation or downregulation of miR-215-3p by transfected plasmids and the protein expression levels of leptin, SLC2A5, Stat3, p-Stat3, JAK2, p-JAK2, VEGF, STYXL1, and FLAD1 were detected by Western blotting **(B)** and were analyzed the expression differences **(C)**. GAPDH was used as a loading control. Representative blots were shown and repeated three times. Bar graphs represent the analysis of the ratios of each protein expression band density to that of GAPDH for nine genes. The ratios were plotted as mean ± SD of three separate experiments. Compared with the control group, “ns” means no statistical difference between means, *P < 0.05; **P < 0.01; ***P < 0.001.

Genes including *Stat3, mTOR, JAK2, VEGF, AKT, Notch-1*, and *PI3K* are tightly related with *leptin* expression and as key genes in the signal transduction. *STYXL1, CUL7, FLAD1*, and *SOX4* are important functional genes associated with the SLC2A5 signaling pathway. After the overexpression or knockdown of miR-215-3p, the mRNA expression levels of *leptin, SLC2A5, Stat3, mTOR, JAK2, VEGF, AKT, Notch-1, PI3K, STYXL1, CUL7, FLAD1*, and *SOX4* were detected by real-time RT-PCR. The results show that the overexpression of miR-215-3p significantly inhibited the mRNA expression of *leptin* and *SLC2A5*, and the mRNA expression of *VEGF, Notch-1, STYXL1*, and *FLAD1* was also significantly decreased, whereas the expression of *AKT, PI3K*, and *SOX*4 was significantly increased. After the knockdown of miR-215-3p, the mRNA expression of *leptin* and *SLC2A5* was significantly enhanced, and the mRNA expression of *VEGF, Notch-1, STYXL1*, and *FLAD1* was also significantly enhanced, whereas the mRNA expression of *AKT, PI3K*, and *SOX4* was significantly decreased. The expression regulation of miR-215-3p had no effect on the mRNA expression levels of *Stat3, mTOR, JAK2*, and *CUL7*. In spite of this, these results could confirm the endogenous targeted regulatory effect of miR-215-3p on *leptin* and *SLC2A5* and affected the mRNA expression level of some related signaling pathway molecules of these two genes (Figure 3A).

### Effect of miR-215-3p on Protein Expression of Genes Related to Leptin and SLC2A5 Signaling Pathway

After the overexpression or knockdown of miR-215-3p, the protein expression levels of leptin, SLC2A5, Stat3, p-Stat3, JAK2, p-JAK2, VEGF, STYXL1, and FLAD1 were detected by Western blotting. The results showed that the overexpression of miR-215-3p significantly decreased the protein expression of leptin, SLC2A5, p-Stat3, p-JAK2, VEGF, STYXL1, and FLAD1, whereas, with the knockdown of miR-215-3p, these proteins levels were markedly elevated. However, the overexpression or knockdown of miR-215-3p has not induced differences in the protein expression levels of Stat3 and JAK2. The results are shown in Figures 3B,C.

### Reduction of lnc-REG3G-3-1 Changed the Growth of Xenograft Tumors in Nude Mice

**Figure 4:**
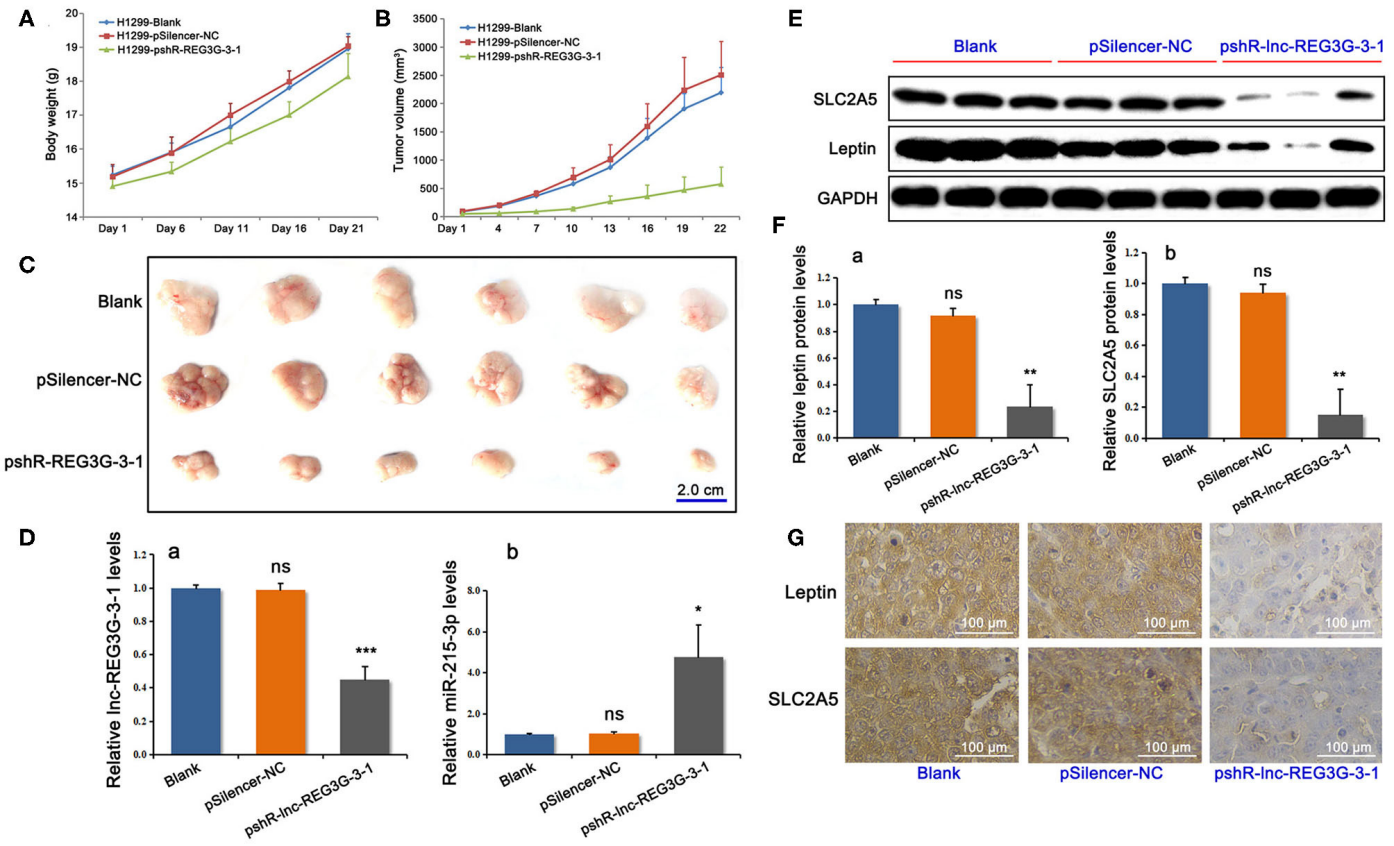
Reduction of lnc-REG3G-3-1 changed the growth of xenograft tumors in nude mice and gene expression levels. The average body weight and tumor volume of each group were obtained during modal mice feeding. The changes in body weight and the growth curve of the tumors are shown in **(A,B)**. The dots of line charts represent the numbers plotted as mean ± SD (*n* = 6). After 21 days, the subcutaneous tumors were removed from each mouse separately **(C)**. After modeling of nude mice xenograft tumors, the subcutaneous tumors were removed and the gene expression of lnc-REG3G-3-1 and miR-215-3p was detected by real-time RT-PCR **(D)**. Western blotting **(E,F)** and immunohistochemistry **(G)** were used to detect the protein levels of leptin and SLC2A5 in tumor tissues. GAPDH was used as loading control. Bar graphs represent the analysis of the ratios of each target band density to that of GAPDH in each group for three genes in tumor tissues. The ratios were plotted as mean ± SD of three separate experiments. Compared with the control group, *P < 0.05; **P < 0.01; ***P < 0.001. For immunohistochemical results, representative photos are shown. Brown staining showed strong protein expression, whereas pale blue staining showed weak protein expression.

The results of the changes in body weight and the growth curve of the transplanted tumors showed that downregulated lnc-REG3G-3-1 remarkably inhibited the growth of the transplanted tumors in model mice (Figures 4A,B). After 21 days, the model mice were sacrificed, and the subcutaneous tumors were removed from each mouse separately. These tumors were weighed, scaled for the measurement of the diameter, and photographed. The results show that the knockdown of lnc-REG3G-3-1 remarkably inhibited the growth of xenograft tumors in nude mice (Figure 4C).

### Reduction of lnc-REG3G-3-1 Changed the Gene Expression Levels in Tissues of Xenograft Tumors in Nude Mice

At the end of feeding and completion of xenograft tumors in nude mice, the subcutaneous tumors were removed. Real-time RT-PCR was employed to detect the gene expression of lnc-REG3G-3-1 and miR-215-3p. The results showed that, compared with the control groups, the lnc-REG3G-3-1 in the knockdown group was significantly decreased in tumor tissues, whereas the miR-215-3p level was remarkably increased (Figure 4D). Western blotting and immunohistochemistry were used to detect the protein levels of leptin and SLC2A5 in tumorous tissue. The Western blotting results for leptin and SLC2A5 protein levels are shown in Figures 4E,F, whereas the immunohistochemical results are shown in Figure 4G. The results show that the reduction of lnc-REG3G-3-1 induced obviously the decrease of the protein expression of leptin and SLC2A5.

## Discussion

LADC is the most common pathological type of lung cancer with a high incidence of BM (21, 22). There is often a lack of effective treatment for BM from LADC with poor prognosis, and 1-year survival is 10% (23, 24). More and more evidence confirms that lncRNAs are closely related to the invasion and metastasis of malignant tumors, including lung cancer (14, 15, 25–27). Studies have shown that the expression of specific lncRNA associated with LADC metastasis is significantly correlated with lymph node metastasis and tumor staging (28). In addition, the lncRNA expression profile in LADC may play an important role in predicting the survival of patients (29). However, the number of lncRNAs was found to be closely related to LADC metastasis is very limited, and the mechanism of function is not completely clear. Therefore, further exploration is still needed.

In this study, microarray gene chip detection found that between LADC tissue and adjacent tissue as well as BM tissue from LADC and adjacent tissue, all have differences in expression of lncRNAs and mRNAs. Thereby, miRNAs related to specific lncRNA were searched through ceRNA mechanism, and their possible target genes were verified. The results show that the expression of lnc-REG3G-3-1 in BM tissues that originated from LADC was significantly higher than that in LADC tissues.

As a hormone secreted by fat cells and playing a key role in promoting angiogenesis, *leptin* is an important factor in the tumorigenesis, metastasis, resistance to apoptosis, and drug resistance of lung cancer and many other cancers by regulating various signal transduction pathways (30). *Leptin* can up-regulate the expression of *VEGF* when overexpressed in lung tumor tissues, thereby promoting the proliferation and remodeling of vascular endothelial cells (31). Studies have found that the *PI3K*/protein kinase B (*PKB*/*Akt*)/*mTOR* signaling pathway is overactivated in NSCLC patients. *Leptin* activates *PI3K*, an important molecule in the mTOR pathway, thereby activating *Akt* and *mTOR*, which are the downstream targets of *PI3K*. The activation of this signaling pathway can regulate the expression of various tyrosine kinase receptors in lung cancer cells, promote the survival and proliferation of cancer cells, and play an important role in the invasion and metastasis of lung cancer (32). *Leptin* expression is significantly increased in various NSCLC cell lines, and its decreased expression level can inhibit cell proliferation and induce apoptosis through the inactivation of *Notch* and *JAK*/*STAT3* signaling pathways, suggesting that downregulating *leptin* may be a new method to block lung cancer metastasis (32).

Fructose metabolism plays an important role in promoting the occurrence and development of malignant tumors. GLUT5, encoded by *SLC2A5*, is a fructose transporter unique to mammalian cells. *SLC2A5* expression determines the fructose absorption and utilization efficiency of LADC cells. The overexpression of *SLC2A5* can promote the proliferation, invasion, and metastasis of LADC cells. *SLC2A5* may be a potential target for treating LADC alone or in combination with other therapies (33). Based on the analysis of GO, KEGG, GenMAPP, BioCarta, and disease database, the results show that reducing *SLC2A5* expression can inhibit the tumorigenesis of LADC paracancerous tissues by inhibiting cell cycle–related gene *CUL7* as well as *STYXL1, SOX4*, and *FLAD1* genes (34). One study confirmed that specifically blocking the transmembrane transport of fructose can significantly inhibit the malignant proliferation, invasion, and metastasis of tumor cells *in vitro* (35). Reverse *SLC2A5* overexpression in LADC tissues may have the biological function of inhibiting tumor metastasis of LADC, providing a possible option for the treatment of LADC.

In this study, it was found that the expression of lnc-REG3G-3-1, *leptin*, and *SLC2A5* in A549 and H1299 cells was significantly increased compared with control cells, and the expression of miR-215-3p was significantly downregulated. These findings aroused our great interest. Experiments confirmed that lnc-REG3G-3-1 could reduce the expression level of miR-215-3p through adsorption and thereby regulate the expression levels of *leptin* and *SLC2A5* genes. Moreover, miR-215-3p with a negative regulatory effect on *leptin* and *SLC2A5* influenced the expression of their signaling molecules, including *VEGF, Notch-1*, p-Stat3, p-JAK2, *STYXL1*, and *FLAD1*. The functional results further verified that inhibiting the expression of lnc-REG3G-3-1 or increasing the expression of miR-215-3p can inhibit the tumor metastasis of LADC. Nude mice xenograft tumor experiments further confirmed that reducing lnc-REG3G-3-1 can inhibit the tumor growth by inducing increased miR-215-3p and decreasing the expression of *leptin* and *SLC2A5 in vivo*.

Collectively, the highly expressed lnc-REG3G-3-1 can reduce miR-215-3p expression and thereby regulate the expression of target genes *leptin* and *SLC2A5*. VEGF, Notch, and JAK/Stat3 signaling pathways and genes, including SOX4, STYXL1, and FLAD1 may all be related to the lnc-REG3G-3-1/miR-215-3p axis in the process of BM from LADC. lnc-REG3G-3-1/miR-215-3p, leptin, and SLC2A5 through regulating signaling pathways may be the mechanism that regulates the biological process of BM in patients with LADC.

## Data Availability Statement

Publicly available datasets were analyzed in this study, these can be found in the NCBI Gene Expression Omnibus (GSE146461).

## Ethics Statement

The studies involving human participants were reviewed and approved by the Medical Ethics Committee of the TUMC. The patients/participants provided their written informed consent to participate in this study. This animal study was reviewed and approved by the Medical Ethics Committee of the TUMC. Written informed consent was obtained from the owners for the participation of their animals in this study. Written informed consent was obtained from the individual(s) for the publication of any potentially identifiable images or data included in this article.

## Author Contributions

CJ, BY, and HZ conceived, designed, and carried out the experiments, analyzed the experimental data, and wrote the paper. CJ guided the experiments and prepared all figures and the table. CJ supervised and directed the project. All authors carried out the partial experiments and analyzed the experimental data. All authors discussed the results and commented on the manuscript and reviewed the manuscript.

## Conflict of Interest

The authors declare that the research was conducted in the absence of any commercial or financial relationships that could be construed as a potential conflict of interest.
