# Supplementary material for: lnc-REG3G-3-1/miR-215-3p Promotes Brain Metastasis of Lung Adenocarcinoma by Regulating Leptin and SLC2A5
Source: Front Oncol. 2020 Aug 12;10:1344. doi: 10.3389/fonc.2020.01344 (PMC7434858; doi:10.3389/fonc.2020.01344)
Supplement: Supplementary file 1 [file Table_1.DOC]

****Supplementary table 1****

**Primer sequences of miRNAs, lnc-REG3G-3-1, leptin, SLC2A5 and β-actin for qRT-PCR**

| **Primer name** | **Primer sequence (5′–3′)** |
| --- | --- |
| *hsa-miR-215-3p-Fwd* | TGCGGTCTGTCATTTCTTTAGG |
| *hsa-miR-4505-Fwd* | TGCGGAGGCTGGGCTGGGACGG |
| *hsa-miR-5787-Fwd* | TGCGGGGGCTGGGGCGCGGGGA |
| *U6-Fwd* | TGCGGGTGCTCGCTTCGGCAGC |
| *Reverse* | CCAGTGCAGGGTCCGAGGT |
| *lnc-REG3G-3-1-Fwd* | GCAAGTGGCAGTTGGCTTAG |
| *lnc-REG3G-3-1-Rev* | TCAGCTGGGAATGGTTGGTG |
| *LEP-Fwd* | TCACACACGCAGTCAGTCTC |
| *LEP-Rev* | GAGGTTCTCCAGGTCGTTGG |
| *SLC2A5-Fwd* | TCTTCCAACGTGGTCCCCAT |
| *SLC2A5-Rev* | AGGGTAGTGGTGAAGGGGTG |
| *β-actin-Fwd* | CGTGACATTAAGGAGAAGCTG |
| *β-actin-Rev* | CTAGAAGCATTTGCGGTGGAC |
