# Supplementary material for: lnc-REG3G-3-1/miR-215-3p Promotes Brain Metastasis of Lung Adenocarcinoma by Regulating Leptin and SLC2A5
Source: Front Oncol. 2020 Aug 12;10:1344. doi: 10.3389/fonc.2020.01344 (PMC7434858; doi:10.3389/fonc.2020.01344)
Supplement: Supplementary file 2 [file Table_2.DOC]

****Supplementary table 2****

**Primer sequences of lnc-REG3G-3-1-3’UTR wt and lnc-REG3G-3-1-3’UTR mut**

| **Primer name** | **Primer sequence (5′–3′)** |
| --- | --- |
| *lnc-REG3G-3-1-3’UTR wt-Top* | PmeI 5´AAACTAGCGGCCGCTAGTTAACTTATGGATGAAATGACAGAT 3´XbaI |
| *lnc-REG3G-3-1-3’UTR wt -Bot* | XbaI 5´CTAGATCTGTCATTTCATCCATAAGTTAACTAGCGGCCGCTAGTTT 3´PmeI |
| *lnc-REG3G-3-1-3’UTR mut-Top* | PmeI 5´AAACTAGCGGCCGCTAGTTAACTTATGGATGAATACTGTCAT 3´XbaI |
| *lnc-REG3G-3-1-3’UTR mut-Bot* | XbaI 5´CTAGATGACAGTATTCATCCATAAGTTAACTAGCGGCCGCTAGTTT 3´PmeI |
