# Supplementary material for: lnc-REG3G-3-1/miR-215-3p Promotes Brain Metastasis of Lung Adenocarcinoma by Regulating Leptin and SLC2A5
Source: Front Oncol. 2020 Aug 12;10:1344. doi: 10.3389/fonc.2020.01344 (PMC7434858; doi:10.3389/fonc.2020.01344)
Supplement: Supplementary file 3 [file Table_3.DOC]

****Supplementary table 3****

**Primer sequences of** pshR-lnc-REG3G-3-1 **for real time RT-PCR.**

| **Primer name** | **Primer sequence (5′–3′)** |
| --- | --- |
| *ShR-lnc-REG3G-3-1-top-1* | 5’GATCCGGATCACATACATGAAGTTGACTCGAGTCAACTTCATGTATGTGATCCTTTTTGA 3’ |
| *ShR-lnc-REG3G-3-1-bot-1* | 5’AGCTTCAAAAAGGATCACATACATGAAGTTGACTCGAGTCAACTTCATGTATGTGATCCG 3’ |
| *ShR-lnc-REG3G-3-1-top-2* | 5’GATCCGATCCTCTTCGTAATGCCAGTCTCGAGACTGGCATTACGAAGAGGATCTTTTTGA 3’ |
| *ShR-lnc-REG3G-3-1-bot-2* | 5’AGCTTCAAAAAGATCCTCTTCGTAATGCCAGTCTCGAGACTGGCATTACGAAGAGGATCG 3’ |
| *ShR-lnc-REG3G-3-1-top-3* | 5’GATCCCAGCTGAACTGTATCCATGAGCTCGAGCTCATGGATACAGTTCAGCTGTTTTTGA 3’ |
| *ShR-lnc-REG3G-3-1-bot-3* | 5’AGCTTCAAAAACAGCTGAACTGTATCCATGAGCTCGAGCTCATGGATACAGTTCAGCTGG 3’ |
