# Supplementary material for: lnc-REG3G-3-1/miR-215-3p Promotes Brain Metastasis of Lung Adenocarcinoma by Regulating Leptin and SLC2A5
Source: Front Oncol. 2020 Aug 12;10:1344. doi: 10.3389/fonc.2020.01344 (PMC7434858; doi:10.3389/fonc.2020.01344)
Supplement: Supplementary file 4 [file Table_4.DOC]

| **Primer name** | **Primer sequence (5′–3′)** |
| --- | --- |
| *Stat3-Fwd* | CAGCGGTAAGACCCAGATCC |
| *Stat3-Rev* | TGGTATTGCTGCAGGTCGTT |
| *mTOR-Fwd* | CAGGGCTTCTTCCGTTCCAT |
| *mTOR-Rev* | TCTTGTTGGCTGCATTGTGC |
| *JAK2-Fwd* | AGCAAGCAAACCAAGAGGGT |
| *JAK2-Rev* | GGGCCATGACAGTTGCTTTG |
| *STYXL1-Fwd* | CTATGAGCGCTTCTCAGGCA |
| *STYXL1-Rev* | TGAATCTTGGGGTCACAGGC |
| *VEGF-Fwd* | TGCCCGCTGCTGTCTAATG |
| *VEGF-Rev* | GCGAGTCTGTGTTTTTGCAGG |
| *AKT-Fwd* | TACGAGATGATGTGCGGTCG |
| *AKT-Rev* | GGCCGTGAACTCCTCATCAA |
| *Notch-1-Fwd* | CTGAATGGCGGGAAGTGTGAA |
| *Notch-1-Rev* | GTTGGTGAGGCAGGCATTGT |
| *PI3K-Fwd* | TTTTGCTGTTCGGTGCTTGG |
| *PI3K-Rev* | CCAAAAGCAGGCCAAACCTC |
| *CUL7-Fwd* | ATTCGAGATGGCAGCAAGGA |
| *CUL7-Rev* | GCCTTGAGGATTCGGACGAT |
| *FLAD1-Fwd* | CTGCTCTCCCCCATTGTCTT |
| *FLAD1-Rev* | GGTGTTGGTGTCCTGAGTGT |
| *SOX4-Fwd* | GCAAACCAACAATGCCGAGA |
| *SOX4-Rev* | ATCTGCGACCACACCATGAA |

****Supplementary table 4****

**Primer sequences of Stat3, mTOR, JAK2, STYXL1, VEGF, AKT, Notch-1, PI3K, CUL7, FLAD1 and SOX4 for real time RT-PCR**
