# Supplementary material for: lnc-REG3G-3-1/miR-215-3p Promotes Brain Metastasis of Lung Adenocarcinoma by Regulating Leptin and SLC2A5
Source: Front Oncol. 2020 Aug 12;10:1344. doi: 10.3389/fonc.2020.01344 (PMC7434858; doi:10.3389/fonc.2020.01344)
Supplement: Supplementary file 10 [file Table_10.DOC]

****Supplementary table 10****

LncRNA expression of gene chip in brain metastases of lung adenocarcinoma and lung adenocarcinoma (Significantly different of Top 8)

| **Expression of lncRNA with significant difference** | | |
| --- | --- | --- |
| **lncRNA ID** | **Regulation** | ***P* value** |
| **ENST00000304425.3** | up | 0.0198 |
| **ENST00000439259.1** | up | 0.0277 |
| RNA96022|RNS_1104_82 | down | 0.0142 |
| ENST00000543559.1 | down | 0.0151 |
| ENST00000515414.1 | down | 0.0227 |
| ENST00000576365.1 | down | 0.0287 |
| ENST00000608770.1 | down | 0.0326 |
| ENST00000448570.1 | down | 0.0368 |
