# Supplementary figures and images for: lnc-REG3G-3-1/miR-215-3p Promotes Brain Metastasis of Lung Adenocarcinoma by Regulating Leptin and SLC2A5
Source: Front Oncol. 2020 Aug 12;10:1344. doi: 10.3389/fonc.2020.01344 (PMC7434858; doi:10.3389/fonc.2020.01344)

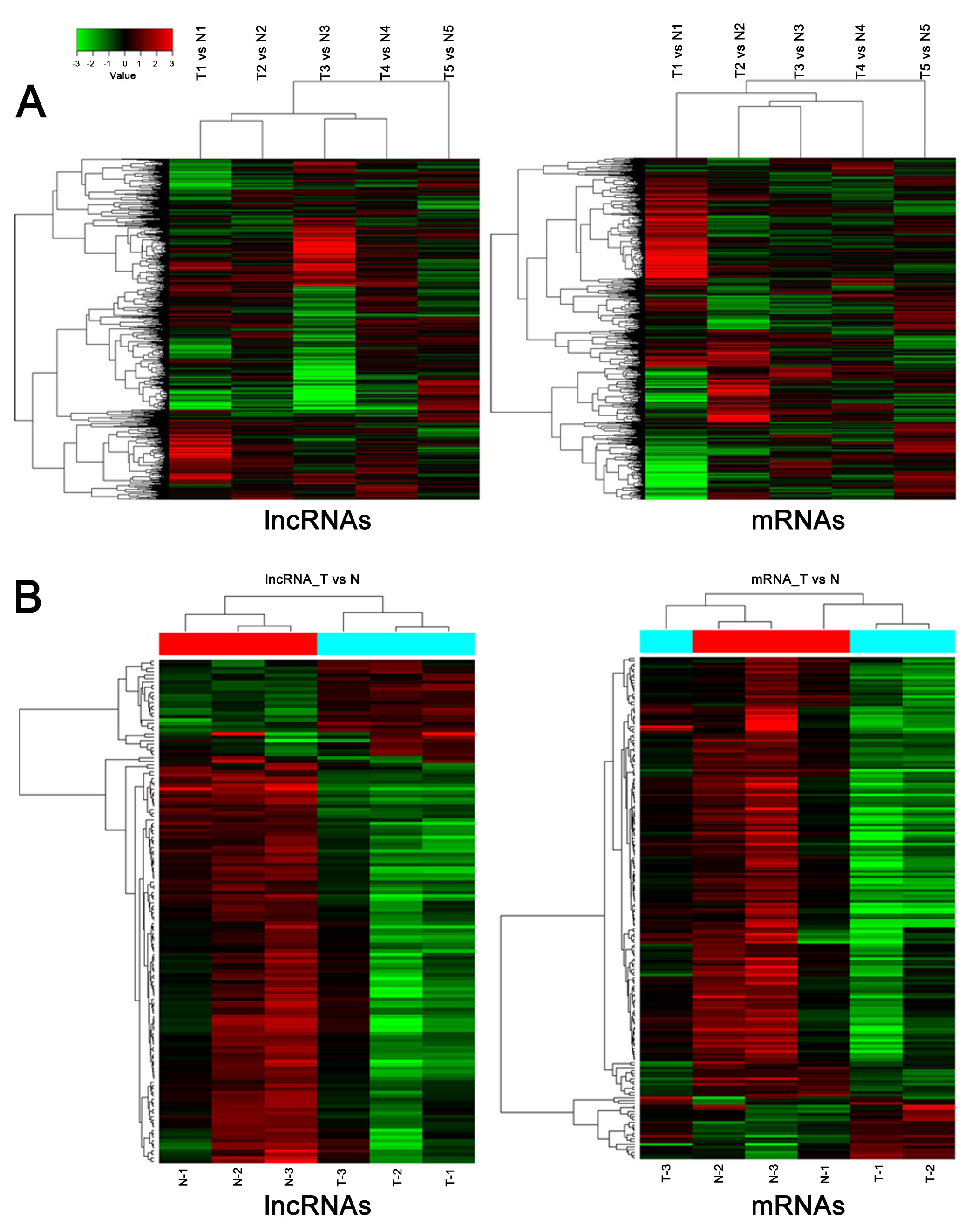

Supplement: Supplementary file 11 [file Image_1.JPEG]

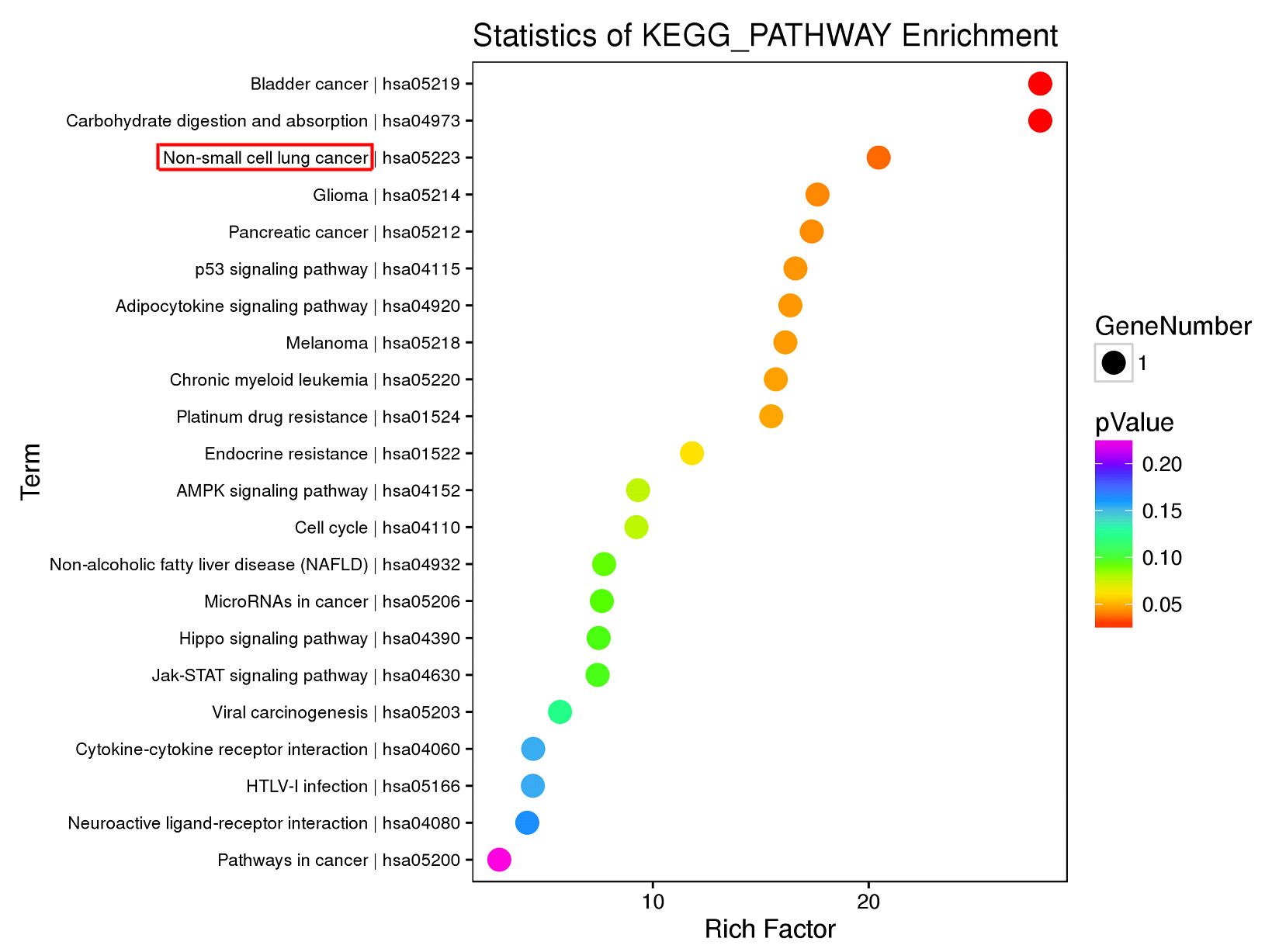

Supplement: Supplementary file 12 [file Image_2.JPEG]

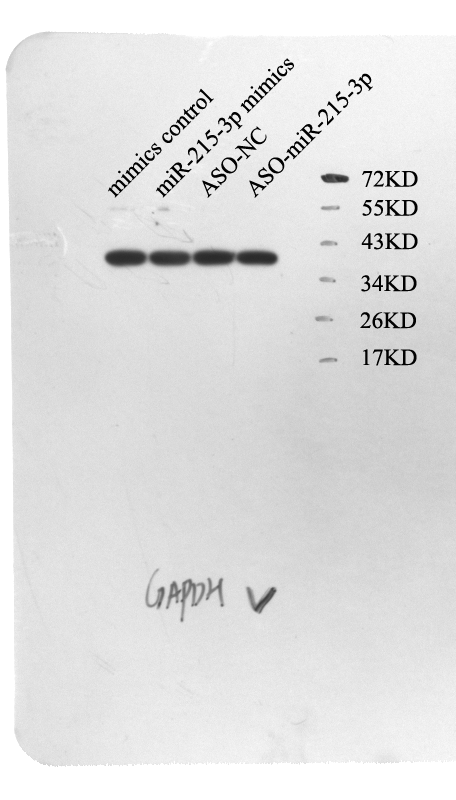

Supplement: Supplementary file 13 [file Data_Sheet_1.ZIP › Western Bloting primary bands/Figure 2 Western Bloting primary bands/1-GAPDH.tif]

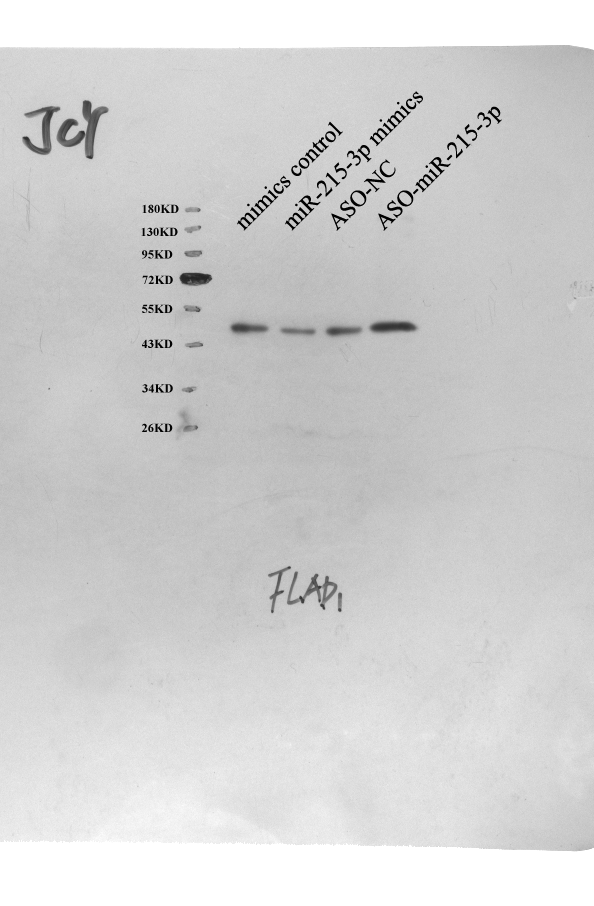

Supplement: Supplementary file 13 [file Data_Sheet_1.ZIP › Western Bloting primary bands/Figure 2 Western Bloting primary bands/10-FLAD1.tif]

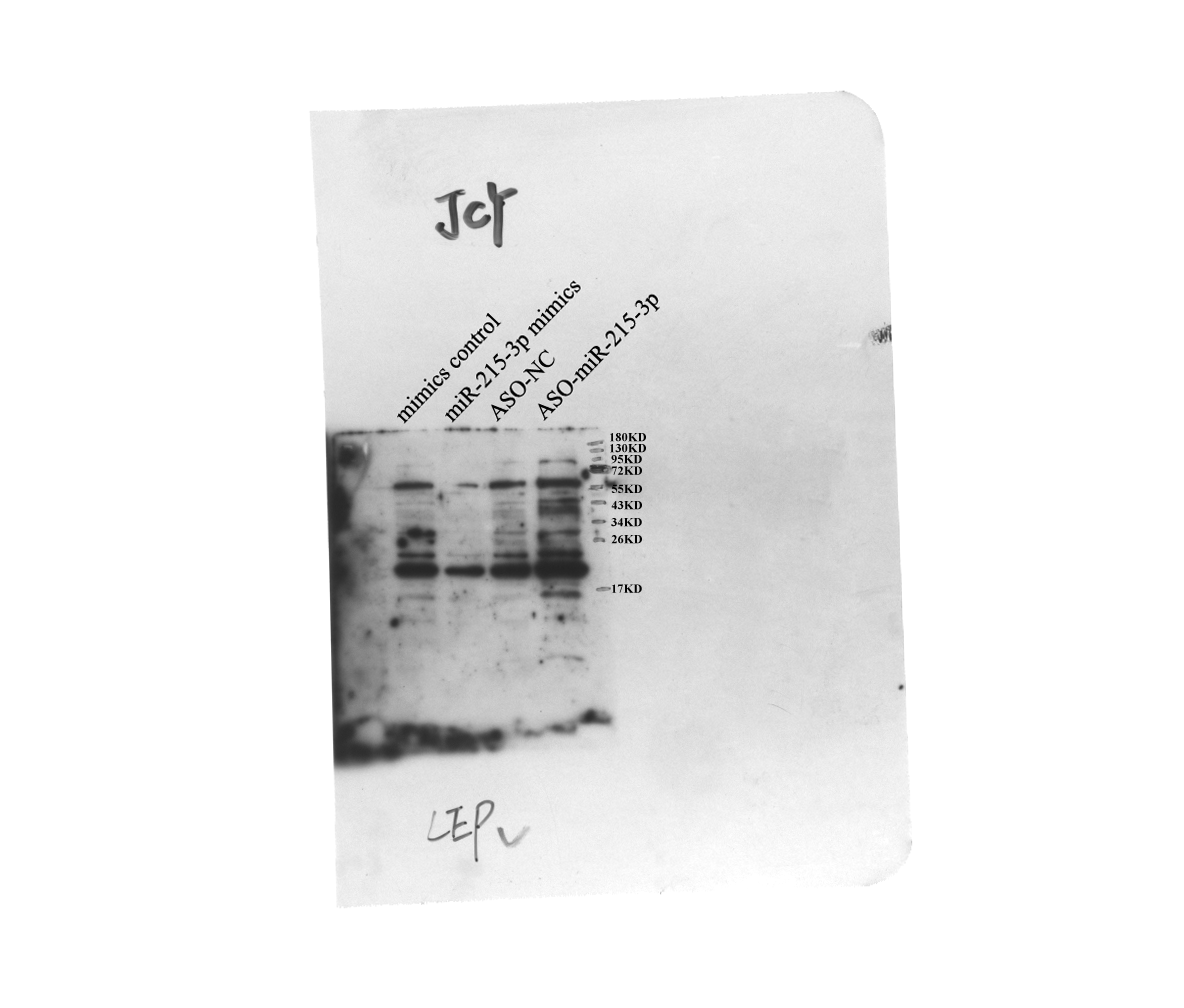

Supplement: Supplementary file 13 [file Data_Sheet_1.ZIP › Western Bloting primary bands/Figure 2 Western Bloting primary bands/2-Leptin (LEP).tif]

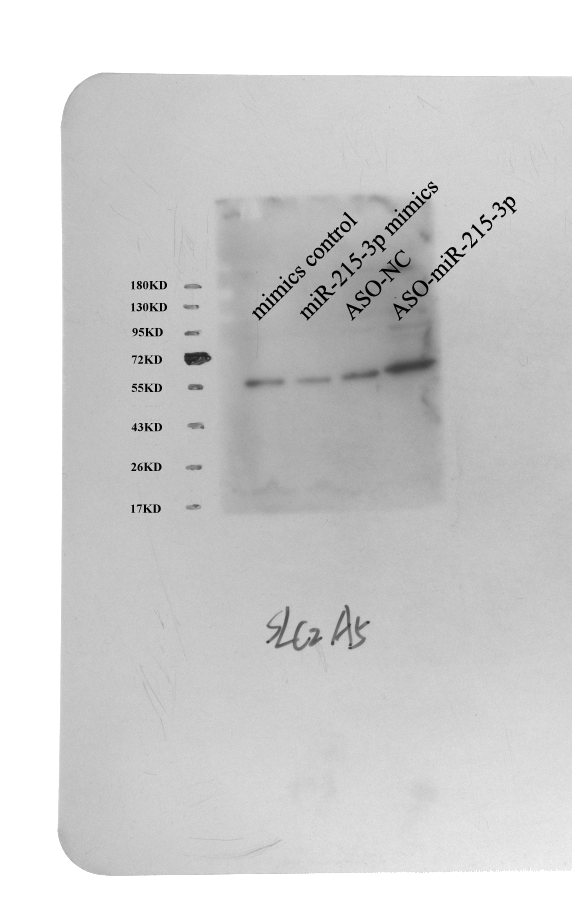

Supplement: Supplementary file 13 [file Data_Sheet_1.ZIP › Western Bloting primary bands/Figure 2 Western Bloting primary bands/3-SLC2A5.tif]

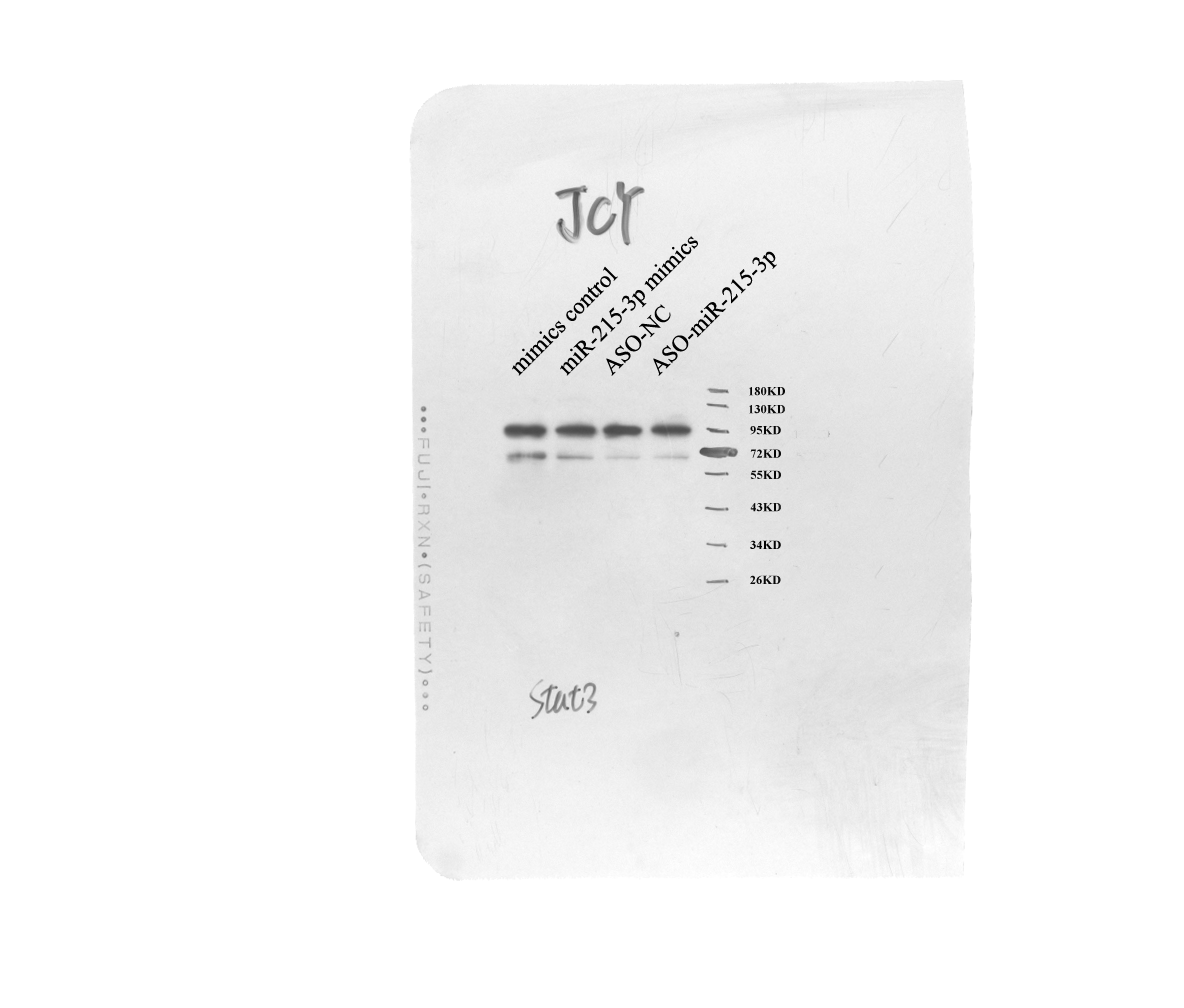

Supplement: Supplementary file 13 [file Data_Sheet_1.ZIP › Western Bloting primary bands/Figure 2 Western Bloting primary bands/4-STAT3.tif]

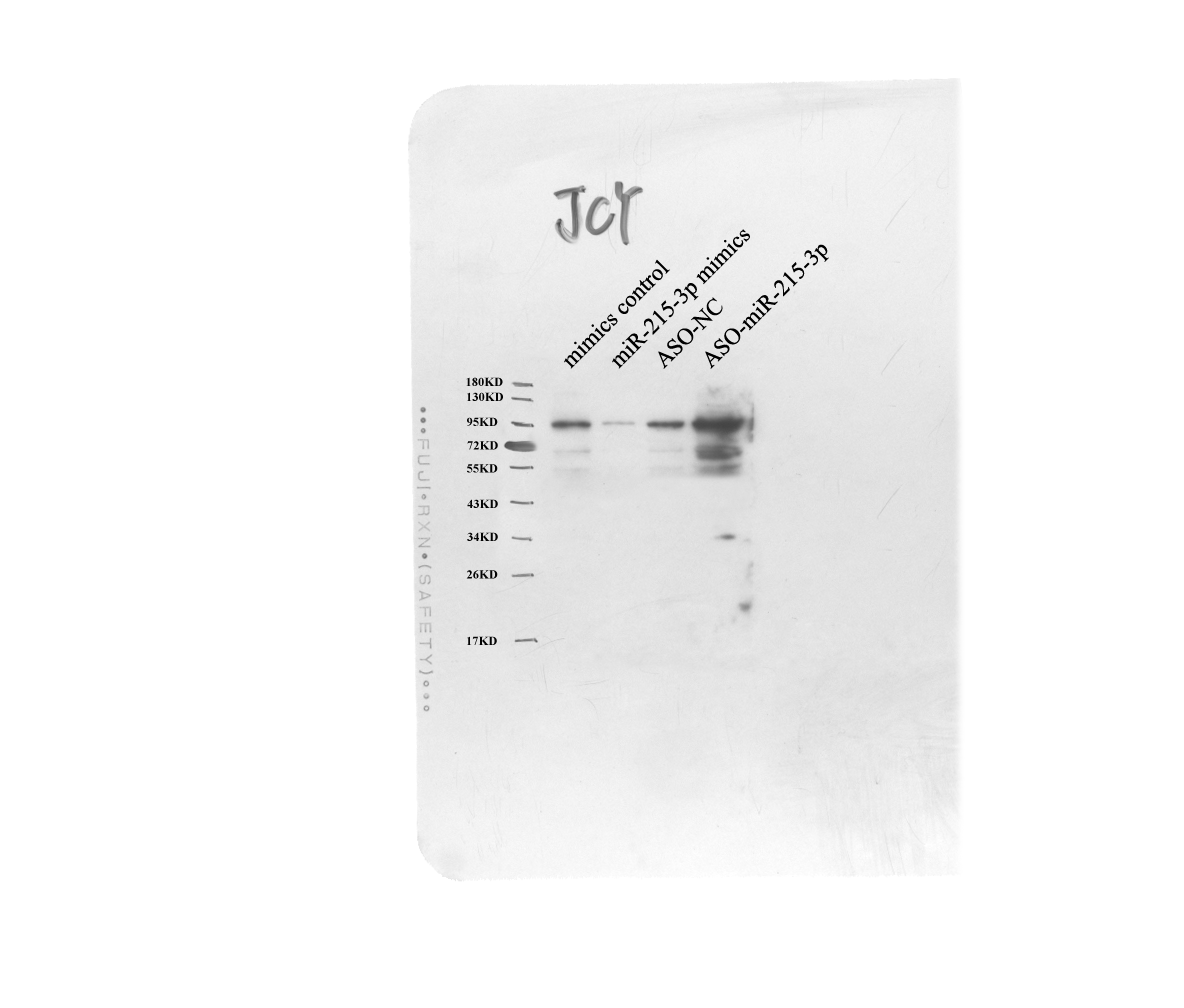

Supplement: Supplementary file 13 [file Data_Sheet_1.ZIP › Western Bloting primary bands/Figure 2 Western Bloting primary bands/5-p-STAT3.tif]

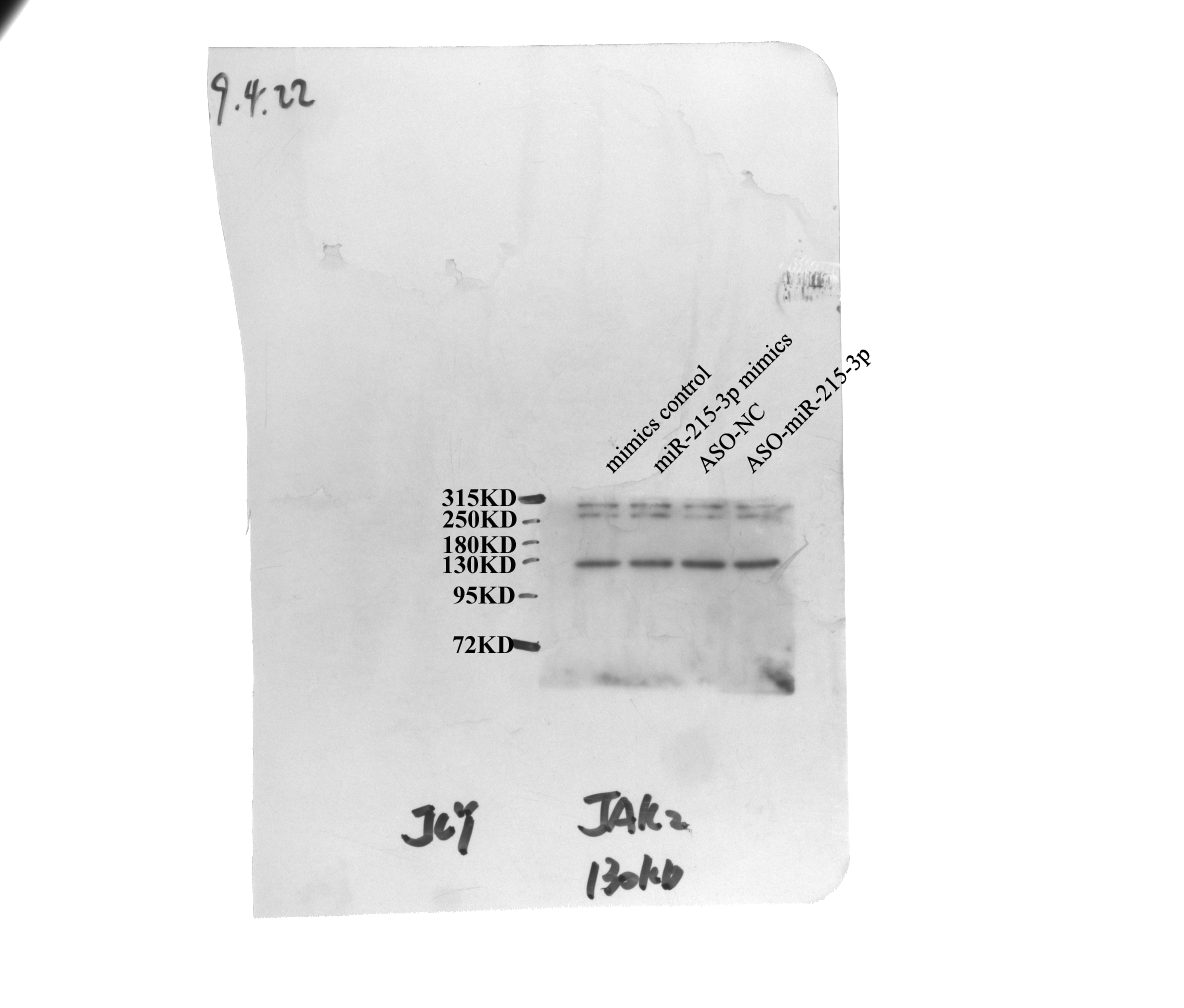

Supplement: Supplementary file 13 [file Data_Sheet_1.ZIP › Western Bloting primary bands/Figure 2 Western Bloting primary bands/6-JAK2.tif]

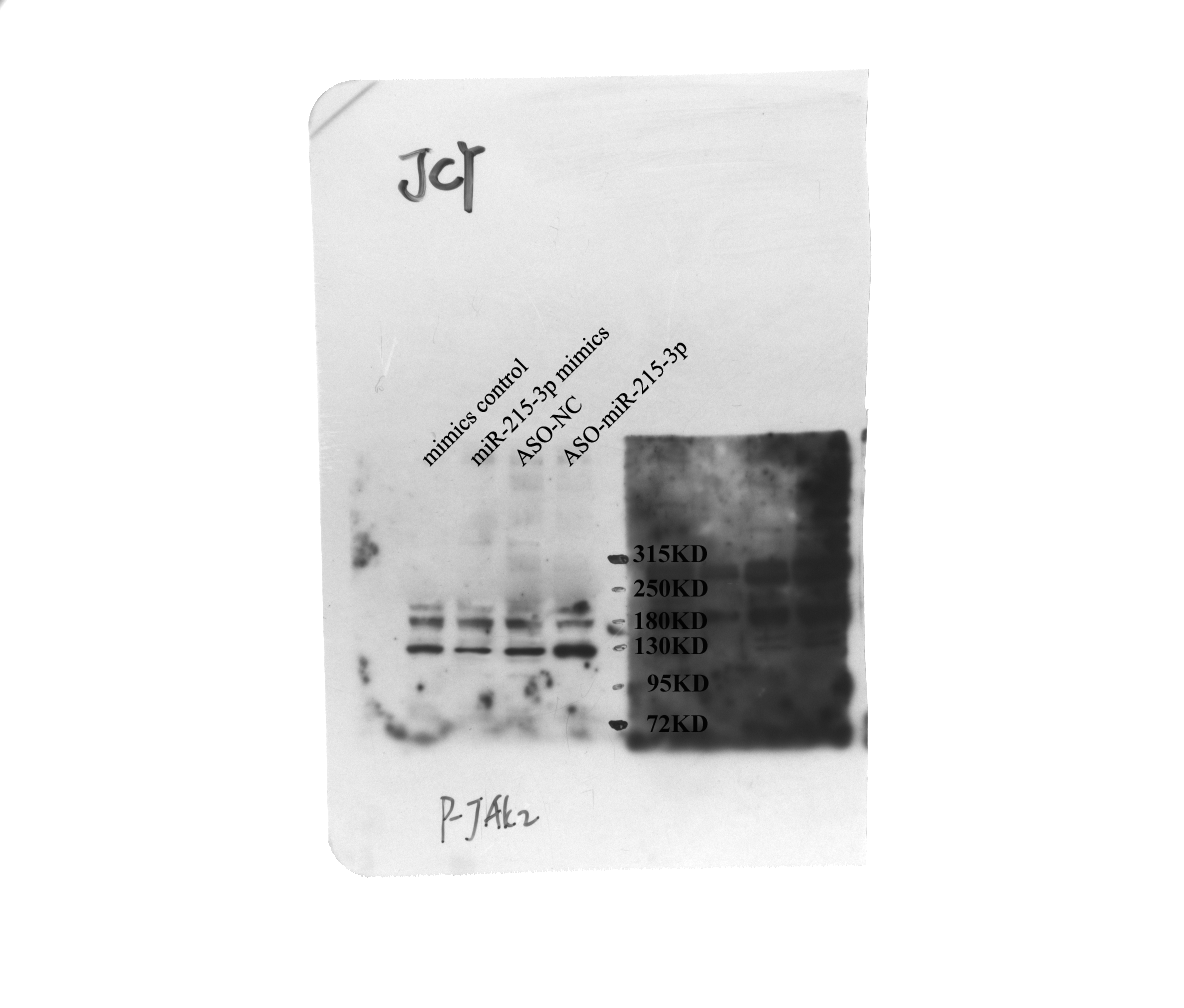

Supplement: Supplementary file 13 [file Data_Sheet_1.ZIP › Western Bloting primary bands/Figure 2 Western Bloting primary bands/7-p-JAK2.tif]

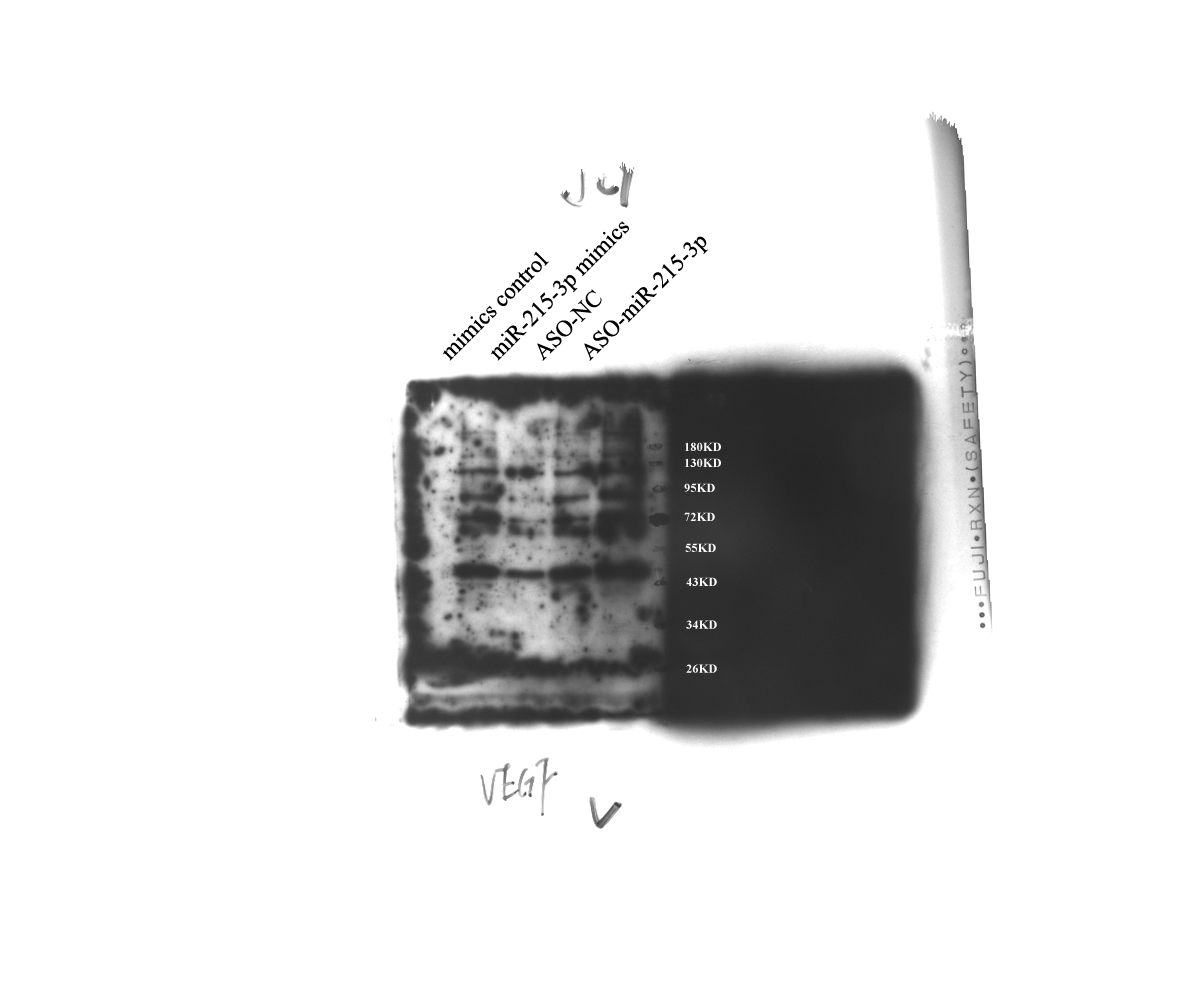

Supplement: Supplementary file 13 [file Data_Sheet_1.ZIP › Western Bloting primary bands/Figure 2 Western Bloting primary bands/8-VEGF.tif]

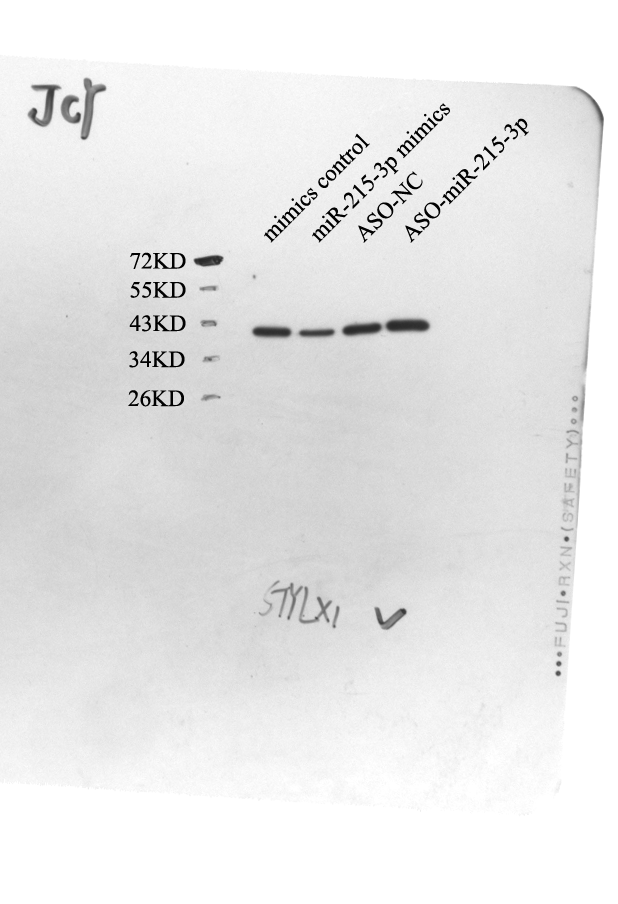

Supplement: Supplementary file 13 [file Data_Sheet_1.ZIP › Western Bloting primary bands/Figure 2 Western Bloting primary bands/9-STYXL1.tif]

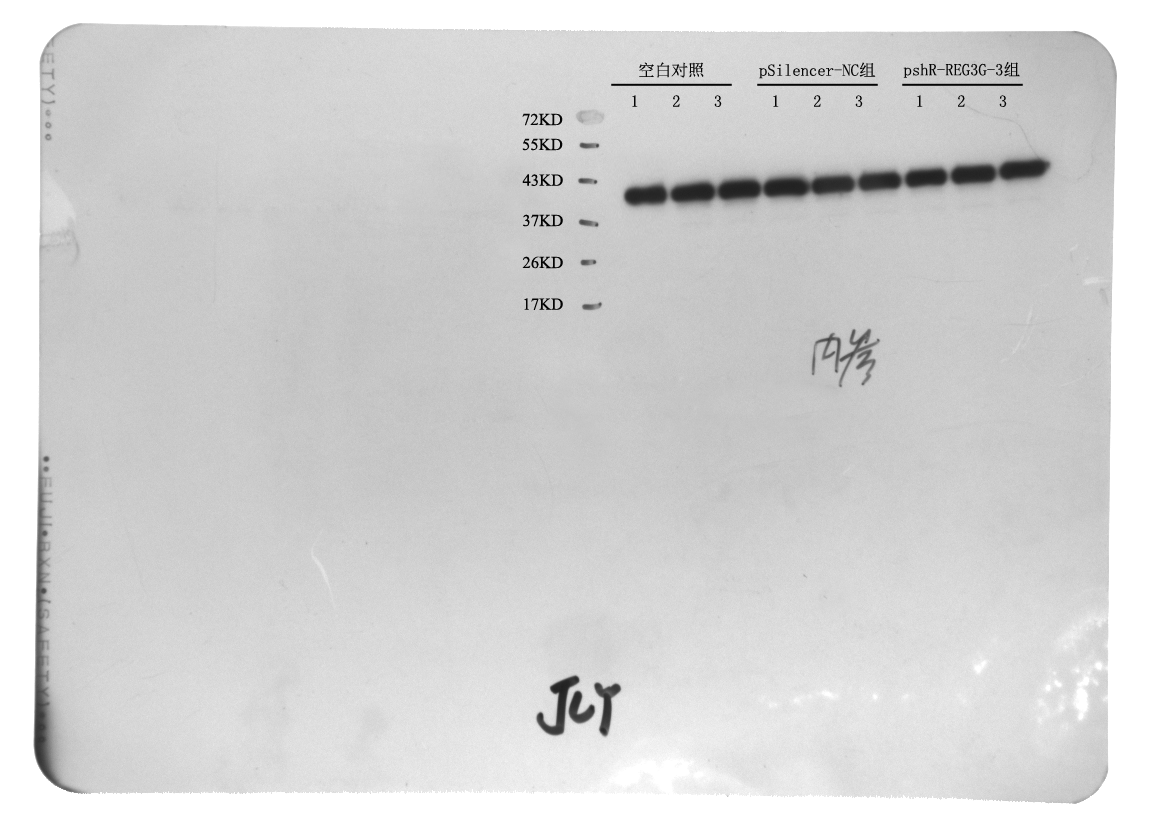

Supplement: Supplementary file 13 [file Data_Sheet_1.ZIP › Western Bloting primary bands/Figure 4 Western Bloting primary bands/1-GAPDH.tif]

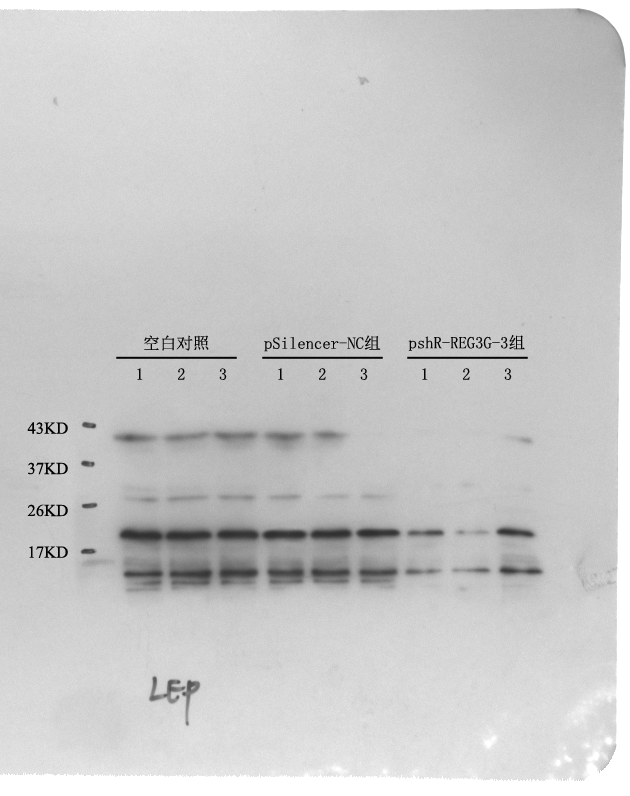

Supplement: Supplementary file 13 [file Data_Sheet_1.ZIP › Western Bloting primary bands/Figure 4 Western Bloting primary bands/2-Leptin (LEP).tif]

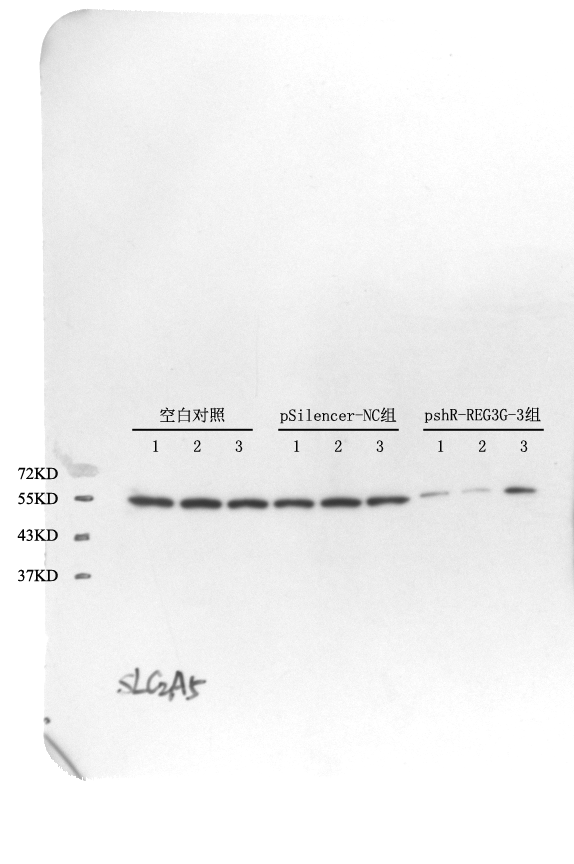

Supplement: Supplementary file 13 [file Data_Sheet_1.ZIP › Western Bloting primary bands/Figure 4 Western Bloting primary bands/3-SLC2A5.tif]
